# MorphoITH: a framework for deconvolving intra-tumor heterogeneity using tissue morphology

**DOI:** 10.1186/s13073-025-01504-x

**Published:** 2025-09-19

**Authors:** Aleksandra Weronika Nielsen, Hafez Eslami Manoochehri, Hua Zhong, Vandana Panwar, Vipul Jarmale, Jay Jasti, Mehrdad Nourani, Dinesh Rakheja, James Brugarolas, Payal Kapur, Satwik Rajaram

**Affiliations:** 1 Lyda Hill Department of Bioinformatics, University of Texas Southwestern Medical Center, Dallas, TX USA; 2 Department of Electrical and Computer Engineering, The University of Texas at Dallas, Richardson, TX USA; 3 Department of Pathology, University of Texas Southwestern Medical Center, Dallas, TX USA; 4 Kidney Cancer Program, Simmons Comprehensive Cancer Center, University of Texas Southwestern Medical Center, Dallas, TX USA; 5 Department of Internal Medicine (Hematology-Oncology), University of Texas Southwestern Medical Center, Dallas, TX USA; 6 Department of Urology, University of Texas Southwestern Medical Center at Dallas, Dallas, TX USA

**Keywords:** Deep learning, Artificial intelligence, Kidney cancer, CcRCC, Tumor morphology, Tumor evolution, Intra-tumor heterogeneity, Histopathology, Digital pathology

## Abstract

**Background:**

Tumor evolution, driven by the emergence of genetically and epigenetically distinct subclones, enables cancers to adapt to selective pressures and become more aggressive, posing a major challenge in oncology. Multi-regional sequencing has been the primary means of studying tumor evolution and the resultant intra-tumor heterogeneity (ITH), but its high cost, resource-intensiveness, and limited scalability have hindered clinical utility.

**Methods:**

Here, we present MorphoITH, a novel framework that aims to infer molecular ITH from routinely collected histopathology slides by quantifying phenotypic diversity. MorphoITH integrates a task-agnostic, self-supervised deep learning similarity measure to capture phenotypic variation across multiple dimensions (cytology, architecture, and microenvironment) along with rigorous methods to eliminate spurious sources of variation.

**Results:**

Applying MorphoITH to clear cell renal cell carcinoma (ccRCC), a disease notably shaped by ITH, we show that it captures clinically significant biological features such as vascular architecture and nuclear grade. MorphoITH also recognizes morphological changes associated with subclonal alterations in key driver genes (*BAP1*, *PBRM1*, *SETD2*). Finally, in a multi-regional sequencing dataset, we find that the morphological trajectories revealed by MorphoITH largely mirror underlying patterns of genetic evolution.

**Conclusions:**

MorphoITH provides a scalable and rigorous approach to quantify morphological ITH, serving as a potential proxy for underlying genetic ITH and tumor evolution. By linking histopathology with genomic insights, it lays the foundation for more refined phenotypic profiling in support of precision oncology.

**Supplementary information:**

The online version contains supplementary material available at 10.1186/s13073-025-01504-x.

## Background

Intra-tumor heterogeneity (ITH), the coexistence of neoplastic cells in distinct molecular and functional states within the same tumor, is a critical challenge for precision oncology [1, 2]. Driven by cancer evolution, ITH contributes to tumor aggressiveness, therapy resistance, and reduced biomarker effectiveness [3]. Multi-regional sequencing has revealed the evolutionary processes underlying ITH [4, 5], but the high cost and logistical constraints of these assays present significant barriers. Even when multi-region sequencing is feasible (e.g., in research studies), it is unclear how one should sample the tumor to efficiently capture ITH and study its evolutionary dynamics, especially in large tumors with high mutation rates where it is impractical to profile the entire tumor [6–8]. There is still much we do not understand about tumor evolution, highlighting a pressing need to explore alternative, scalable modalities for characterizing ITH.

An attractive alternative is to use tissue morphology from hematoxylin and eosin (H&E) stained histopathological slides that are already routinely used by pathologists to characterize tumors. Tissue morphology is used for diagnosis and for assessing tumor aggressiveness [9–11] and likely reflects phenotypic expression of molecular states in tumor cells. Under a microscope, extensive information about tumors can be appreciated, including tumor cell features, such as nucleolar prominence, variations in nuclear shape, and cytoplasmic characteristics. In addition, pathologists assess how tumor cells self-organize to form tumor architectures and how they relate to their microenvironment [12]. While the sheer scale and complexity of these features (~ 10^5^ cells per slide) make quantification difficult [13], computational approaches offer an opportunity to systematically characterize ITH. Indeed, we and others have shown that applying deep learning (DL) approaches to histopathologic slides can deconvolve ITH by identifying driver mutational states, molecular subtypes, and diagnostically distinct areas [14, 15]. However, past work has focused on predefined aspects, rather than the totality of ITH.

**Fig. 1:**
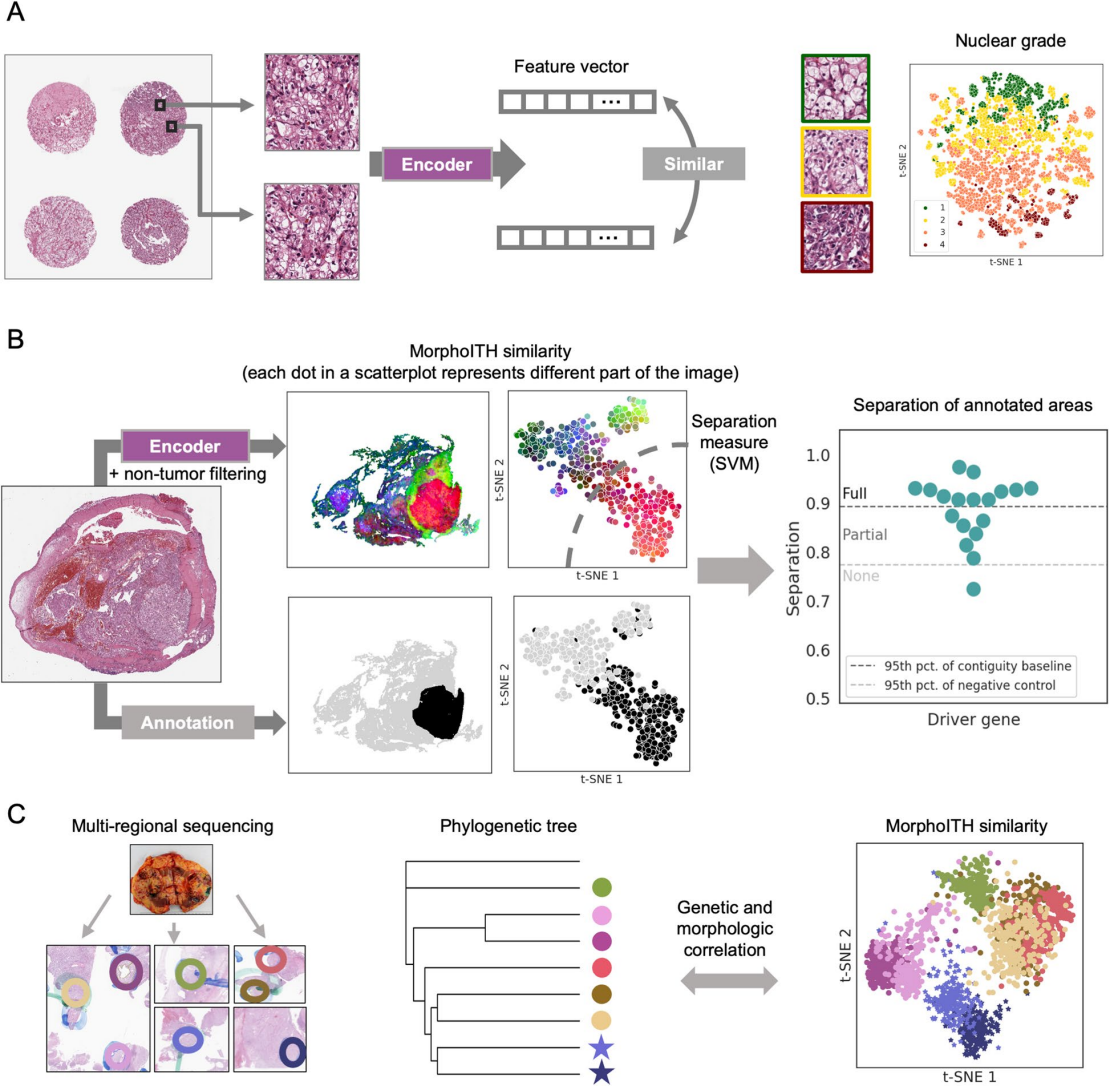
Overview of MorphoITH, a framework for characterizing morphological similarity from histopathological slides. **A** Encoder development: MorphoITH employs a self-supervised deep learning model to generate feature vectors representing morphological similarity. For this application, the model was trained using input patches from the same tissue microarray (TMA) cores with the objective that they yield similar feature vectors. Despite being self-supervised, the resulting features are biologically meaningful, e.g., image patches (points in scatter plot) of similar nuclear grade (colors) tend to have similar MorphoITH profiles. **B** Separability analysis framework: MorphoITH incorporates statistical analyses to assess how well morphological heterogeneity (top row: false color depiction of MorphoITH profiles for WSI ID: 19390 from WSI-3 cohort) relates to heterogeneity in a given parameter of interest (bottom row: in black shows *BAP1* loss status). This “separability” is quantified and interpreted relative to randomized baselines that account for spatial autocorrelation, categorizing it as fully separable (full), partially separable (partial), and not separable (none). **C** Application to tumor evolution: Morpho ITH-derived morphological similarity is used to explore relationships with genetic tumor evolution. The panel illustrates this for Patient A from a multi-regional sequencing dataset (WSI-1 cohort), showing sampled H&E areas (left; a matched gross kidney image for this specific patient is not available), their corresponding positions on a genetically derived phylogenetic tree (middle; stars denote metastatic samples), and a t-SNE visualization of their MorphoITH patch features (right)

Here, we introduce MorphoITH, a functionally unbiased framework to deconvolve ITH from histopathologic slides. We prototype this approach in clear cell renal cell carcinoma (ccRCC), a cancer type that is renowned for its genetic ITH with key driver mutations known to be subclonal [7, 13, 16]. Our approach is designed to address two key challenges. First, quantifying biologically meaningful morphologic differences and differentiating them from inconsequential fluctuations. We address this using a self-supervised DL model; for its training within this study, we leveraged the assumption that in a tissue microarray (TMA), areas in close physical proximity are more likely to be functionally similar (Fig. 1A). Second, reducing inference of spurious statistical correlations caused by the tendency of spatially proximal regions to be similar [17]. To address this, we incorporate statistical controls thatprovide a more reliable evaluation of the relationship between morphology and molecular readouts (Fig. 1B).

We validated MorphoITH in several ways. First, we showed its ability to capture biologically meaningful tissue types, vascular architectures, and nuclear grade changes. Then, we applied MorphoITH to evaluate the effect of key driver gene mutations in ccRCC (*BAP1*/*PBRM1/SETD2*), demonstrating that morphological heterogeneity reflects genetic heterogeneity and can guide efficient sampling strategies. Finally, using multi-regional sequencing data from three patients (26 samples total), we explored how tumor evolution relates to morphological ITH, and observed a surprising level of concordance given the unsupervised nature of MorphoITH, while also identifying notable expectations that may, for example, reflect convergent evolution (Fig. 1C). These findings position MorphoITH as a scalable, unbiased tool for understanding the interplay between morphology and genetic heterogeneity, with implications for improving tumor sampling and advancing precision oncology.

## Methods

### Datasets

The datasets used in this study, including their key characteristics and primary purpose, are summarized below, and detailed comprehensively in Additional file 1: Table S1. All datasets consist of H&E-stained slides from formalin-fixed paraffin-embedded (FFPE) tissue blocks obtained through retrospective studies. No patients were prospectively enrolled for the purposes of this study. The slides were scanned at either 20 × or 40 × resolution, but all analyses were performed at 20 × (0.5 microns per pixel), with images downsampled as needed. For analyses requiring image patches, 224 × 224-pixel patches were extracted, with overlap allowed.

1. TMA Training: This dataset was used to train the model, and to select model hyperparameters on a pretext task (with the dataset split into training and heldout testing sets). The full training dataset has 10 tissue microarray blocks, 785 cores total, from 421 ccRCC patients surgically treated at UTSW and representing all stages of the disease [18–20].
2. TMA Validation: This dataset was used as an independent ccRCC TMA cohort to validate additional retrieval tasks. It contains 95 patients (with no patient overlap with the TMA Training set) treated for high-stage ccRCC at UTSW [20, 21]. The dataset consists of 3 blocks, 183 cores total, that had their core-level nuclear grades assigned by pathologists.
3. WSI-1: This cohort consists of 41 ccRCC WSIs from UTSW (UTSeq Data [22]). The dataset served two purposes: (a) to evaluate MorphoITH’s ability to detect ITH in grade and architecture, a pathologist annotated 16 WSIs that contained at least two regions with distinct nuclear grades, and 26 WSIs that contained at least two regions with different vascular architectures; (b) to explore the relationship between morphology and tumor evolution, we used multi-region whole-exome sequencing data from three patients (26 samples across 18 WSIs). Sampling sites were manually selected (average area ~ 6.6 mm^2^) to be morphologically uniform (see Additional file 1: Table S1 for more information).
4. WSI-2: This cohort of 10 ccRCC WSIs from UTSW (UTSW CD31 Re-stain [22]) had two applications: (a) to evaluate MorphoITH’s ability to detect ITH in grade and architecture, a pathologist annotated 4 WSIs that contained at least two regions with distinct nuclear grades, and 7 WSIs that contained at least two regions with different vascular architectures; (b) for an analysis of handcrafted features from tumor areas of whole slide images (10 WSIs).
5. WSI-3: This cohort of 1268 ccRCC WSIs from Mayo Clinic [23, 24] (each representing a single patient), that had known driver gene status of wild-type (WT) or loss (as assessed by immunochemistry (IHC) assays done on serial sections) was utilized as an input for heterogeneity score calculations. A subset of cases that exhibited areas with both loss and WT driver gene status within the same slide (18 *BAP1*, 9 *SETD2*, and 9 *PBRM1*) were additionally annotated for their localized (focal) loss.
6. TCGA KIRC: This cohort of 444 ccRCC WSIs from the Pan Cancer Atlas study [25] (each representing a single patient) was used to assess separation of tumor from non-tumor. Additionally, data from 348 of these patients was used for heterogeneity and survival analysis (see Additional file 1: Table S1).

### MorphoITH encoder: training and selection

We designed a pretext retrieval task to guide the development of the MorphoITH encoder, optimizing its deep learning architecture, backbone, and input type (details of the explored configurations are in Additional file 1: Fig S1A). For architectures, we tested the following: (1) BYOL [26–28], which does not require negative examples for training, as well as (2) Triplet (with TripletMarginLoss [29]) and (3) MoCov2 [30], which do. For BYOL and Triplet, backbones were either a base Vision Transformer (ViTb [31]) or ResNet50 [32], both pretrained on ImageNet [33], while for MoCov2 we preserved its original ResNet50 backbone. Training was conducted with a batch size of 32 and the Adam optimizer (learning rate of 3e − 4). Output of the last linear layer of the models was 1,000-dimensional. Input patches were augmented using HED- and color-jitter, random gaussian blur, elastic deformation, and random rotation [34]. Additionally, the extent of color saturation was randomly reduced using a multiplier. The training task involved feeding the models pairs of patches that were either an augmented pair of: the same patch (input type: same), two different patches from the same TMA core (input type: close), or a combination of both types (input type: combined). If the training framework required negative examples, these were defined as patches from different TMA cores.

To select the optimal encoder configuration from these candidates, the TMA Training cohort was divided into fixed folds: one for training (approximately 2/3 of the cohort), and one for testing using the pretext retrieval task (1/3 of the cohort). This task assessed how often a patch’s nearest neighbors (by cosine distance in feature space) originated from the same TMA core (see Additional file 1: Fig. S1B, C for best epochs’ results). The best-performing configuration was selected as the MorphoITH encoder and subsequently retrained on the entire TMA Training cohort.

To enable a comparison of our MorphoITH encoder with a publicly available foundation model in downstream tasks (detailed in "Impact of Encoder Choice" in "Results"), we also employed this same pretext retrieval task to select a representative, high-performing candidate. From several models evaluated (UNI [35], Virchow [36], GigaPath [37], CONCH [38], RetCCL [39]), UNI (v1) was selected due to its strong performance on the pretext task and its computational manageability (Additional file 1: Fig. S15A).

### Whole-slide images inference

We performed inference on patches tessellated across each WSI (100 pixels step size) to generate a grid of outputs from two models: (1) the MorphoITH encoder, which generated 1,000-dimensional feature vectors for each patch, and (2) a pretrained in-house region classifier, which predicts patches as tumor, normal, stroma, blood, necrosis, immune, or background. For the WSI-1 and TCGA KIRC cohorts, we additionally used GrandQC to discard areas with foreign objects and pen marks [40].

The in-house region classifier utilizes a ViTb architecture and was trained on 224 × 224 pixel patches extracted from pathologist-annotated ground-truth tissue regions on 124 ccRCC WSIs from UTSW. To test MorphoITH’s ability to distinguish tumor versus non-tumor regions, we used the region classifier as ground truth (after smoothing its activation outputs using a Gaussian filter with kernel size 3). For all subsequent tasks, we focused purely on the MorphoITH profiles from tumor regions (“non-tumor filtering”). Tumor areas with fewer than 100 tessellated patches were discarded.

### Separability analysis

We first obtained ground truth assignments of areas on WSI images into different classes. These assignments were based either on pathologist annotations of the areas on WSIs (for architecture, grade, and driver mutation status, with the latter manually transferred over from a corresponding IHC slide), or on the output of our in-house region classifier. Given such ground truth assignment of areas from two classes (e.g., tumor vs. non-tumor), we created a measure to assess their separability based on MorphoITH features.

First, the 1,000-dimensional MorphoITH outputs were reduced to 10 dimensions using PCA. Then, a linear support vector machine (SVM) classifier with class balancing was trained using 50% of the patches to distinguish the two classes, and the accuracy of the model on the held-out 50% was used as the separability measure. In cases where there was more than one class present in a slide, we iteratively focused on each pair of classes.

In order to account for spatial autocorrelation, we generated two sets of control ground truth classes: (a) contiguity baseline: by randomly creating circular annotations within the regions, while maintaining the area size of each class, and (b) negative control: by splitting each contiguous region for a class in half and giving each half a different label. We generated 10 random control ground truths for each annotated class pair in a WSI, calculated their separability scores, and pooled across all WSIs. We then calculated the 95th percentile for each of these distributions.

A (non-control) separability score was assessed as indicating the following: (a) full separability if it was above 95th percentile of contiguity baseline; (b) partial separability if it was between 95th percentile of the two controls; and (c) no separability if it was below the 95th percentile of negative control.

### Similarity visualization

As 1,000-dimensional outputs of MorphoITH on WSI are difficult to interpret, we developed a visualization (Fig. 1B, colors on top row of middle panel) that assigns similar colors to patches in a WSI with similar MorphoITH features. To achieve this, we performed PCA of the MorphoITH features, and for each patch took the first three principal component loadings, scaled them to the [0, 1] range and used them to define colors in an RGB color scheme.

### Morphological descriptors

MorphoITH’s morphological similarity measure was compared to a set of independent handcrafted morphological descriptors: (1) nuclear sizes calculated from StarDist nuclear detection [41, 42], (2) vasculature density from a vascular model that outputs vascular masks [22], (3) eosin intensity for non-nuclear pixels, obtained by converting RGB to HED color space using the nuclear masks as a guide. For each pair of patches, similarity in the handcrafted descriptors was defined as the absolute difference in their values. In contrast, MorphoITH’s similarity was computed as the cosine similarity between their 1,000-dimensional feature vectors. For each slide, we chose the 100 most similar pairs of patches based on each of the 4 similarity measures (three handcrafted descriptors and MorphoITH features). To ensure similarity was not due to overlapping cells, we required that each patch pair be separated by at least 10 patch widths (after tessellation) within the WSI. We then compared the similarity scores of these selected pairs to those of 100 randomly selected pairs, evaluating their relative rank within the similarity distribution.

### Spatially constrained clusters

To obtain spatially contiguous regions of similar morphology within each WSI, we applied agglomerative hierarchical clustering to the MorphoITH patch feature vectors. This process utilized the following: (1) Ward’s linkage method; (2) a spatial connectivity matrix, derived from patch adjacency to ensure clusters were spatially contiguous; and (3) a predefined number of clusters (*N*) per slide. While *N* = 10 was used for the primary analysis, we also evaluated *N* = 5, 15, and 20 to assess the dependence of key findings on cluster number. For each spatial cluster, a single representative feature vector was determined by identifying the patch feature vector closest (by cosine distance) to the mean feature vector of that cluster.

### Single slide heterogeneity score

To quantify intra-slide morphological heterogeneity, we developed a per-slide heterogeneity score. This score was calculated from the *N* = 10 spatially constrained morphological clusters described above. First, we computed all pairwise cosine distances between the representative feature vectors of these clusters, excluding clusters with areas below the 5th or above the 95th percentile of the cluster area distribution within the cohort, to minimize outlier influence. The per-slide heterogeneity score was then defined as an exponentially weighted average of these inter-cluster distances, to give greater weight to larger morphological dissimilarities. Specifically, to calculate the weights, we normalized the distances for each slide to the [0, 1] range, and the exponential of these distances was used as a weighting factor for the corresponding unnormalized distances.

We applied this heterogeneity score to two analyses:

1. WSI-3 cohort: To test the relationship between morphological and genetic heterogeneity, we compared the heterogeneity score between slides with focal loss of *BAP1*/*PBRM1*/*SETD2* and those with uniform (non-focal) gene status.
2. TCGA KIRC cohort: To assess the prognostic value of the score, we examined its relationship to Progression-Free Survival (PFS) using Kaplan–Meier analysis (stratifying patients by the median heterogeneity score) and multivariate Cox proportional hazards regression (with nuclear grade and the heterogeneity score as covariates).

### Overlap analysis

To test the hypothesis that MorphoITH-derived morphological clusters respect known genetic boundaries, we performed an overlap analysis. This involved quantifying the spatial concordance between two partitions of a WSI’s tumor region from the WSI-3 cohort: (1) loss/WT for *BAP1*/*PBRM1*/*SETD2* as described in the WSI-3 cohort description above, and (2) *N* = 10 spatially constrained morphological clusters generated by MorphoITH. Specifically, for each morphological cluster, we calculated the proportion of its area that overlapped with the loss region. To minimize the impact of potential inaccuracies in registering IHC-defined boundaries to the H&E slide, a narrow boundary zone (derived by dilating the annotated boundary between WT and loss regions with a 5 × 5 pixel kernel) was excluded from area calculations. We defined a morphological cluster to be “split” between WT/loss regions if its overlap with the loss region was in the range of 0.2–0.8.

To determine if MorphoITH clusters were split less often than expected by chance given the spatial configuration of WT/loss regions, we compared the proportion of split MorphoITH clusters to the proportion of split clusters derived from our control segmentations (i.e., contiguity and negative controls). This baseline comparison was repeated 10 times per slide with randomly generated control clusters. Fisher’s exact test was used to assess whether the proportion of split MorphoITH clusters was significantly lower than that observed for the controls.

### Morphology guided sampling

Our goal was to use morphological heterogeneity detected by MorphoITH to propose regions within a WSI for multi-region sequencing that better capture genetic heterogeneity. We tested this approach on slides from WSI-3, with intra-slide genetic ITH in *BAP1*/*PBRM1*/*SETD2*, where the goal was to extract punches with loss and WT with a minimum set of areas profiled. Given a budget of *N* areas to profile, our recipe is to identify the *N* most morphologically distinct regions based on MorphoITH and profile representative areas from each. This was achieved by performing spatially constrained clustering (as described above) with *N* clusters and choosing a representative patch for each. If for the *N* chosen representative profiles their corresponding labels included both loss and wild-type, we considered the heterogeneity capture a success and quantified the overall performance across slides in terms of the fraction of successes. Similar to the “Overlap analysis”, we discarded areas labeled as “at boundary”. Random sampling was performed by randomly choosing a representative patch from the tumor area. To test whether MorphoITH improved the success rate over random sampling for the same number of punches, we used Fisher’s exact test.

### Somatic variant calling from whole-exome sequencing

The WSI-1 cohort includes whole-exome sequencing (WES) of FFPE tissue samples from 3 ccRCC patients, each represented by multiple tumor regions and one adjacent normal site. Raw reads FASTQ data were processed using the SCHOOL pipeline [43] to trim reads, align to human reference genome GRCh38 (hg38), and call somatic variations from paired tumor and adjacent normal samples. The pipeline used stringent criteria to output only high-confidence variants, requiring single-nucleotide variations and small indels to be reported by three callers: Mutect2 (GATK version 4.1.4.0) [44], freebayes (version 1.2.0) [45] and Strelka2 (version 2.9.10) [46]. Somatic allelic copy number variations (CNVs) were called on the same paired tumor and normal WES samples using the FACETS (version 0.6.2) and FACETS-suite (version 2.0.8) [47] R packages. Chromosome-arm-level gain or loss was called when > 50% of the chromosome arm showed copy number gain or loss.

### Phylogenetic trees

Both somatic synonymous and non-synonymous variations, as well as chromosome arm-level copy number variations, were used to construct phylogenetic trees. For each patient, we identified the entire set of observed variant events across all samples and used it to construct a variation matrix (variant × sample). In this matrix, events associated with mutations were assigned as 0 for wild-type and 1 for mutants (VAF > 0). For arm-level CNVs, events were specified as −1/0/1 (loss/neutral/gain). The column corresponding to the normal sample had all 0s. Pairwise Hamming distances between samples based on this matrix were used as our “genetic distance” and used to construct a phylogenetic tree based on the nearest-neighbor algorithm, which was further refined by nearest-neighbor interchange (NNI) rearrangement optimized for maximum parsimony. Finally, the tree was re-rooted to start from the normal sample. Trees were visualized in Fig. 4 in ultra-metric format (tips of the tree are equidistant from the root), while in Additional file 1: Fig. S11 the trees were visualized as phylograms (tree lengths are proportional to the amount of change). The order of the parallel branch splits was rotated manually (while respecting the phylogenetic relationships in the tree) to better match the corresponding t-SNEs. Alterations likely to be important (COSMIC Cancer Gene Census curated list of oncogenes [48] and TRACERx Renal signature CNVs [7]) were marked on the corresponding tree branches. We only displayed alterations next to a branch if they were as follows: (a) in coding regions, (b) found in all samples along that branch, and (c) absent in samples diverging in other directions.

### Morphological similarity for evolution

For each patient, MorphoITH features for all samples were extracted either without normalization (Fig. 4) or after stain normalization using Macenko [49] or Vahadane [50] schemes, matched to the color distribution of 250 randomly sampled patches from the patient (Additional file 1: Fig. S14). The features were plotted as t-SNEs with a high perplexity value (500) to emphasize global structure and highlight the trajectories of morphological evolution. For further analyses, mean representations per sample were calculated and compared by measuring cosine distances between pairs of samples (“MorphoITH distance”). To check for significant correlation between morphological and genetic distances, we used Mantel’s test [51].

## Results

### Model development and validation

Our goal is to develop a model that, given an input image patch, summarizes its morphological essence in a feature vector (Fig. 1A), which can be used for phenotypic deconvolution of tumor heterogeneity and the study of its biological underpinnings (Fig. 1B, C). However, as no two image patches are identical, we need the features to focus on the functionally relevant aspects of tissue morphology and ignore less consequential differences. For a general-purpose morphological descriptor, we cannot focus on specific functional classes or morphological traits but must learn the relevant phenotypes in an unbiased fashion. We reasoned that patches of nearby tissue are likely to be functionally similar and sought to perform self-supervised training such that patches from the same TMA core had similar feature vectors, relative to those from different cores. To decide between several machine learning alternatives in training such a model (Additional file 1: Fig. S1A), we used a pretext retrieval task to make this selection: how often the nearest neighbors of a patch in feature space came from the same TMA core on an unseen dataset (held out portion of TMA Training; “MorphoITH encoder: training and selection” in the “Methods” section). The highest accuracy was achieved using a BYOL training strategy and a Vision Transformer encoder (Additional file 1: Fig. S1B, C). This configuration was selected as the MorphoITH encoder and is used in all subsequent analyses except in the section “Impact of encoder choice”.

We sought to evaluate how well MorphoITH performs as a more global similarity measure by comparing patches from different cores. Both in a nearest neighbor analysis and a global t-SNE representation, we found that when patches were considered similar but originated from different cores, they tended to have similar morphology, such as the same nuclear grade (Fig. 1A t-SNE plot, Additional file 1: Fig. S2A–C; TMA Validation in the “Methods” section). Notably, regions of heterogeneity within a single core (e.g., tumor vs. stromal patches) were accurately distinguished as distinct phenotypes (Additional file 1: Fig. S2D). Taken together, these results suggest that our approach reliably captures morphologic differences.

### Framework for quantifying morphologic distinguishability between regions

**Fig. 2:**
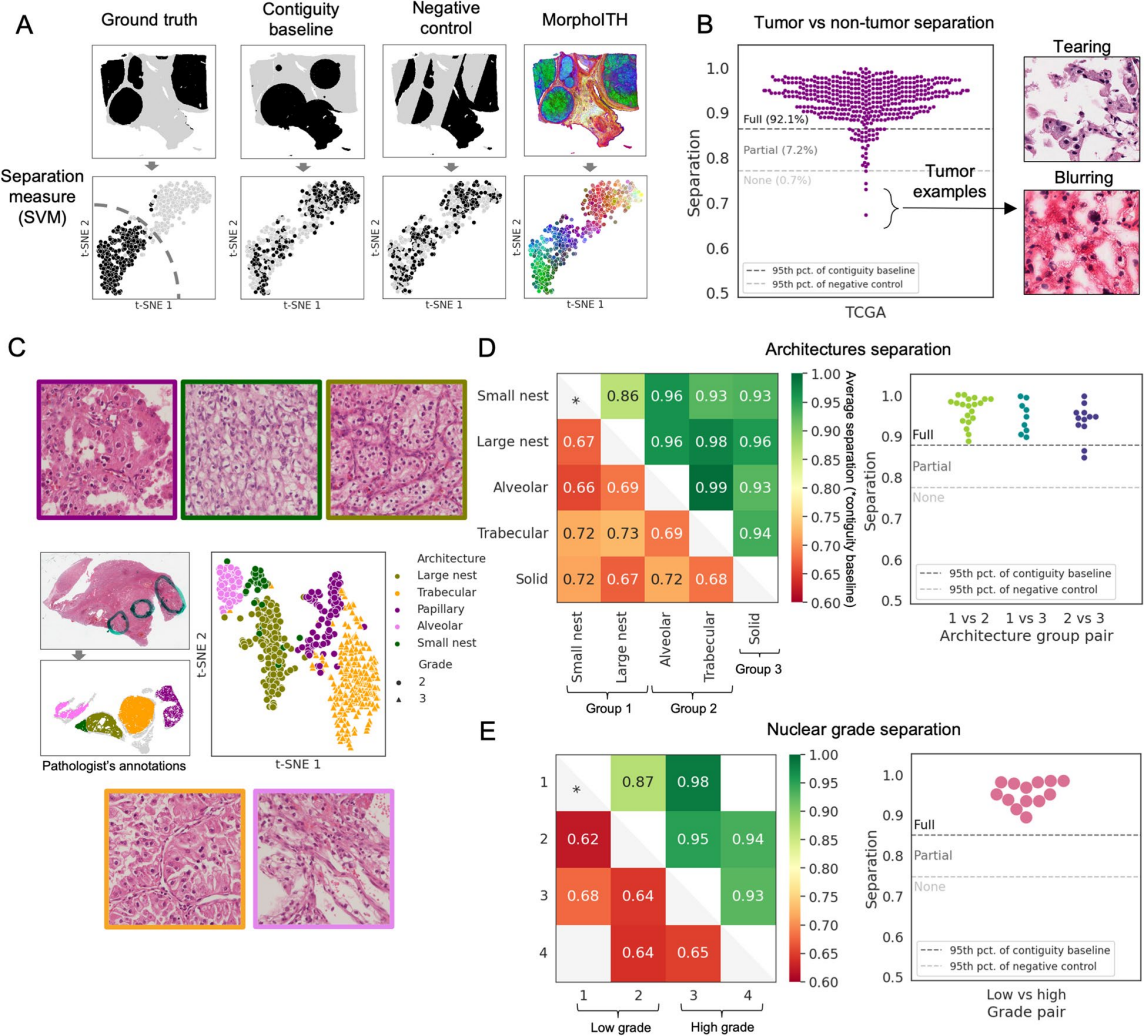
MorphoITH recognizes variance in tissue types, vascular architectures, and nuclear grade in ccRCC. **A** Separability Framework: given ground truth annotation of property of interest (top left: tumor vs. non-tumor for TCGA WSI ID: TCGA-G6-A5PC-01Z-00-DX1), we developed an approach to measure how morphologically distinct these are. MorphoITH features were derived for all patches in a slide (pseudo color on right, and t-SNE on bottom row). From these features, a separation score (bottom left; SVM accuracy) is calculated to quantify the morphological distinction between the annotated regions. To control for spatial autocorrelation, this separability score is interpreted relative to two empirically derived null distributions. The contiguity baseline, built by randomly adding circles with areas corresponding to those of ground truth annotations, validates our approach against random contiguous annotations. Negative control ensures that variance within annotations is comparable to that across them. Separability scores are constructed for multiple random locations of these baselines, and exceeding the 95th percentile of the contiguity and negative baselines defines full/partial and no separation. **B** Separation (*y*-axis) of tumor vs. non-tumor tissue across whole slide images (individual points) from TCGA KIRC. Points were assigned as full/partial/no separation relative to tumor region masks (as explained in **A**). Two examples of tumor areas of samples that were not separable with image artifacts (tearing, blurring) are shown. **C** An example of a whole slide image with areas differing in nuclear grade and vascular architecture from the WSI-1 cohort. Example images of the slide’s architectures are visualized with frame colors corresponding to the legend. We plot a t-SNE with 600 randomly sampled patches, which shows how MorphoITH-derived feature space separates both grades and architectures. **D**, **E** Separation of **D** vascular architectural classes and **E** nuclear grades, assessed across multiple slides (subset of WSI-1 and WSI-2) as compared to contiguity baseline and negative control. Heatmaps show average separation of the ground truth annotations (upper half) and average contiguity baseline control (lower half). Strip-plots include comparisons of separation (*y*-axis) between pairs of annotations, now with coarser labels (*x*-axis): architecture class 1 (small and large nests, bleeding follicles) vs. class 2 (alveolar, trabecular) vs. class 3 (solid), and low grade (1–2) vs. high grade (3–4). Each point represents a comparison between a pair of annotations within a slide

Next, we extended this framework to analyze ccRCC whole slide images (WSIs). To quantify how well two regions differing in a predefined parameter (e.g., molecular state) are distinguishable based on their morphology, we developed a separability metric based on the performance of a naïve linear classifier trained to distinguish MorphoITH profiles (Fig. 1B; “Separability analysis” in the “Methods” section). A key challenge in interpreting this separability is that physically proximal regions tend to be similar both morphologically and molecularly. To address this, we established two baseline distributions (Fig. 2A) for comparison: (1) a contiguity baseline that preserves the spatial structure of tissue regions, and (2) a true negative control where the ground truth classes are randomly split to simulate intra-class morphological variability. Based on these baselines, we classified separability as follows: (a) fully separable if it exceeded the 95th percentile of the contiguity control distribution, (b) partially separable if it fell between the 95th percentiles of the contiguity and negative control distributions, and (c) not separable otherwise.

We first validated this framework by testing the ability of MorphoITH to distinguish tumor and non-tumor regions (black vs. gray areas in Fig. 2A: ground truth). We used a sliding window to generate 1,000-dimensional morphological feature vectors across patches in a slide (“Whole-slide images inference” in the “Methods” section). To visualize spatial patterns of morphological similarity, we used a pseudo-color visualization (“Similarity visualization” in the “Methods” section) such that areas with similar MorphoITH feature vectors are similar in color (Fig. 2A, last column). This approach distinguished tumor and non-tumor areas (Additional file 1: Fig. S3A: tissue types form their own clusters, Fig. S3B: two separate clusters in the tumor region), suggesting that MorphoITH clustered morphologically similar regions. More quantitatively, on a cohort of 444 TCGA KIRC slides, our framework correctly separated tumor and non-tumor regions in 92% of cases (Fig. 2B, Additional file 1: Fig. S4A). In 7% of the cases, separability was partial, likely due to ambiguous tissue classification (e.g., regions consisting of tumor interspersed within stroma; Additional file 1: Fig. S4B). The remaining less than 1% represent failures due to staining artifacts or tissue damage (Fig. 2B, Additional file 1: Fig. S4C). Thus, MorphoITH was able to distinguish between normal and tumor areas within the same slide.

Next, we evaluated MorphoITH’s ability to resolve clinically relevant morphologic features. We focused on tumor architecture and nuclear grade. We categorized recurring architectural patterns, identified based on the spatial arrangement of blood vessels, into 3 prognostic classes. Class 1: small and large nests, bleeding follicles; Class 2: alveolar, trabecular, papillary; and Class 3: solid [13, 52, 53]. At the cellular level, nuclear grade is a key clinical feature of ccRCC with significant prognostic implications. According to nuclear grade, ccRCC was classified into low (grade 1–2) and high (grade 3–4) grade tumors. Nuclear grading is based on tumor cell nuclear size and nucleolar prominence [54]. For our experiments, a pathologist in training (VP) manually partitioned tumor regions based on architecture and grade, with variations in both grade and architecture often seen within the same slide (Fig. 2C; WSI-1 and WSI-2 cohorts in the “Methods” section). MorphoITH fully separated areas of different architectural classes in 95% of cases, with small and large nests occasionally grouped together due to their morphological similarity [52] (Fig. 2D average separation score—upper half: for the ground truth annotations; lower half: for contiguity baseline control, Additional file 1: Fig. S5A). Similarly, areas of low and high grade within the same slide were fully separable (Fig. 2E, Additional file 1: Fig. S5B).

Interestingly, although MorphoITH was not trained using predefined morphological descriptors such as nuclear size or vascular density, it implicitly captures them (Additional file 1: Fig. S6, “Morphological descriptors” in the “Methods” section). Essentially, areas considered similar by MorphoITH are also similar by all the other measures, but the converse is not always true (changes detected by MorphoITH may not be detected by individual descriptors). This highlights MorphoITH’s ability to combine multiple morphological aspects into a unified similarity metric. In summary, MorphoITH offers a general-purpose measure of morphological similarity that captures a wide range of clinically relevant as well as other morphology features.

### MorphoITH detects subclonal changes in the status of key driver genes

**Fig. 3:**
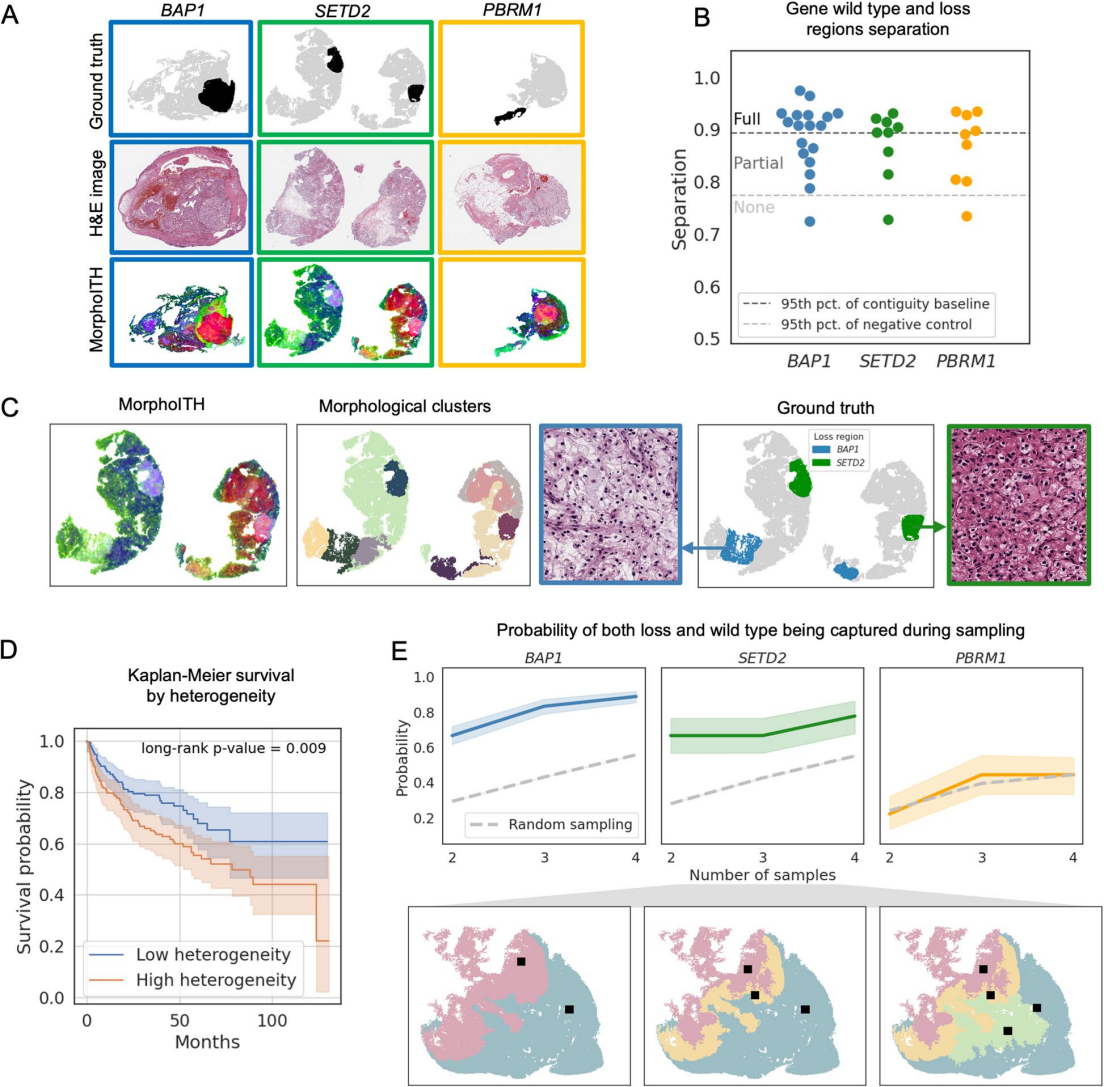
MorphoITH detects morphological changes in regions with loss of driver gene function. **A** Example tumor areas exhibiting intra-slide heterogeneity, with distinct regions in wild-type (WT) and loss-of-function states for key driver genes. We show three driver genes (columns) and their ground truth based on annotations from custom IHC assays, H&E images, and MorphoITH output visualization using pseudo-colors that indicate morphological similarity (rows). From left to right, the IDs of WSIs are as follows: 19097, 19390, 19462 (WSI-3 cohort). **B** Separability scores for distinguishing WT versus loss regions for *BAP1*, *SETD2*, and *PBRM1* across 36 slides from the WSI-3 cohort (each dot represents one slide). 58% of cases have a full separation, 33% partial separation, and 8% no separation. **C** The relationship between MorphoITH-based spatially constrained morphological clusters and genotype in a case (from the WSI-3 cohort, ID: 19390) with heterogeneity in both *BAP1* and *SETD2*. Examples images of morphologies of *BAP1* and *SETD2* loss are shown with frame color corresponding to the legend. **D** Kaplan–Meier analysis of progression-free survival (PFS) in the TCGA KIRC cohort (*N* = 318 patients). Patients were stratified by the median per-slide MorphoITH heterogeneity score (log-rank *p* = 8.5e − 3). **E** Morphology-guided sampling. Bottom: illustration of the strategy for *N* sampling sites; *N* morphological clusters are identified, and one representative patch per cluster is selected as a sampling site (WSI ID: 18625, WSI-3 cohort). Top: probability of capturing both loss and WT driver gene states for *BAP1* (*p* = 6.2e − 8), *SETD2* (*p* = 3.2e − 3), and *PBRM1* (*p* = 0.53) as a function of *N* using MorphoITH-guided (solid lines, mean with 95% CI) versus random sampling (dashed gray line). *P*-values from Fisher’s exact test

Next, we explored the relationship between morphological heterogeneity, as assessed by MorphoITH, and genetic ITH. Rather than predicting the driver gene status within histopathology slides [14], we compared areas within a slide differing in driver gene status and asked if there were *any* differences in morphology, regardless of whether these were specific to that driver gene. For these experiments, we used matched slides with H&E (WSI-3 cohort in the “Methods” section) and immunohistochemistry (IHC) staining that reported the state of three key ccRCC driver genes (*BAP1*, *SETD2*, and *PBRM1*). We focused on slides with heterogeneity in these genes. Based on the corresponding IHC, a pathologist annotated “wild-type” (WT) and “loss” regions on H&E images. As expected, for each of these genes, we observed morphologic changes at the boundaries between wild-type and areas of loss (Fig. 3A). Next, we deployed MorphoITH more systematically across 34 slides with ITH in one of these genes. 58% showed full separability (distinct morphologies for WT vs. loss), 33% partial separability (sub-regions deviating from the broader genotype-associated morphology), and 8% no separability (Additional file 1: Fig. S7A). Among the three genes, we found the strongest morphological effects were seen in *BAP1*, followed by *SETD2* and *PBRM1* (Fig. 3B).

Interestingly, even within genetically defined WT or loss regions, we often observed morphologically distinct sub-regions, suggesting local variation unaccounted for by these driver genes. To investigate this further, we developed an automated approach that partitions slides into spatially constrained morphologic clusters using MorphoITH features (“Spatially constrained clusters” in the “Methods” section). A compelling illustration is shown in Fig. 3C: applying this clustering to an annotated case with subclonal loss of both *BAP1* and *SETD2* (as opposed to single-gene loss visualized earlier), we found that distinct morphological clusters closely corresponded to each of these gene loss regions.

Motivated by such examples, we hypothesized that if indeed these MorphoITH-derived clusters represent underlying “subclones”, they would be contained entirely within either the WT or loss areas, rather than spanning genetic boundaries. To test this, we classified clusters as “split” or not based on their spatial overlap with annotated WT/loss regions (Additional file 1: Fig. S7B; “Overlap analysis” in the “Methods” section). Then, we tested how frequently our clusters were split, relative to randomly generated ground-truth regions derived from our contiguity and negative control baselines. For *BAP1* and *SETD2*, the MorphoITH clusters largely respected the genetic boundaries, a finding consistent across a broad range of cluster numbers (Additional file 1: Fig. S7C). In contrast, for *PBRM1*, while the number of MorphoITH clusters split across the WT/loss boundary was low, this tendency did not reach statistical significance relative to its contiguity baseline. This was likely because slides with *PBRM1* heterogeneity often consisted predominantly of either WT or loss regions (Additional file 1: Fig. S7D), meaning even spatially contiguous control clusters rarely spanned both. Taken together, these findings, particularly the boundary adherence of MorphoITH clusters to *BAP1* and *SETD2* loss regions, support the idea that morphologically distinct areas often correspond to underlying genetic subclones.

To further understand how genetic ITH relates to morphological ITH, we developed a per-slide heterogeneity score based on the morphological clusters (“Single slide heterogeneity score” in the “Methods” section). We used this score to analyze 1268 WSIs from WSI-3, comparing slides with both loss and WT regions for the three driver genes (focal cases) to those with homogeneous gene status. While most slides exhibited some morphological heterogeneity (Additional file 1: Fig. S8A), possibly due to variation in other genes, slides with ITH in *BAP1* showed a higher degree of morphological heterogeneity than those with homogeneous gene status, with a similar, albeit weaker, effect observed for *PBRM1* and *SETD2* (Additional file 1: Fig. S8B). These findings therefore suggest that distinct genetic subclones actively shape the tumors’ morphological landscape, contributing significantly to its overall heterogeneity.

To evaluate the prognostic significance of this morphologic ITH, we calculated per-slide heterogeneity scores for the TCGA KIRC dataset (Additional file 1: Table S1) and correlated them with various clinical variables. We observed that the heterogeneity score increased with nuclear grade (Additional file 1: Fig. S8C) and metastasis (M stage Additional file 1: Fig. S8D), suggesting that morphologic ITH increases during cancer progression. Next, we stratified patients into low- and high-heterogeneity groups based on a median split of the score. Kaplan–Meier analysis revealed that the high-heterogeneity group had significantly worse progression-free survival (PFS) compared to the low-heterogeneity group (Fig. 3D, log-rank *p*-value = 8.5e − 3 for number of clusters *N* = 10; results remain significant for *N* = 5, 15, 20, Additional file 1: Fig. S8E), with the score being especially prognostic at lower grades (Additional file 1: Fig. S8F). Furthermore, in a multivariate Cox proportional hazard regression including both nuclear grade and the heterogeneity score, the score emerged as an independent predictor of PFS (*p* = 0.019). Thus, even though a single slide may not completely reflect the extent of morphological heterogeneity within a tumor (Additional file 1: Fig. S8G), our per-slide MorphoITH heterogeneity score quantifies a prognostically significant aspect of morphological ITH that increases with tumor progression and offers predictive power for PFS beyond nuclear grade in ccRCC.

Finally, following the idea that morphologically distinct areas are in distinct molecular states, we wondered whether morphology-guided sampling could help in more efficient capture of molecular ITH. Specifically, we propose a morphologically guided approach (Fig. 3E) where we partition slides into a specified number of most morphologically distinct areas and capture one sample from each (“Morphology guided sampling” in the “Methods” section). We found that sampling based on morphology, captured the genetic heterogeneity for *BAP1* and *SETD2 *much more efficiently than random sampling, more than doubling the likelihood of capturing both a WT and loss area with two samples.

### Morphology similarity reflects underlying evolutionary trajectories

**Fig. 4:**
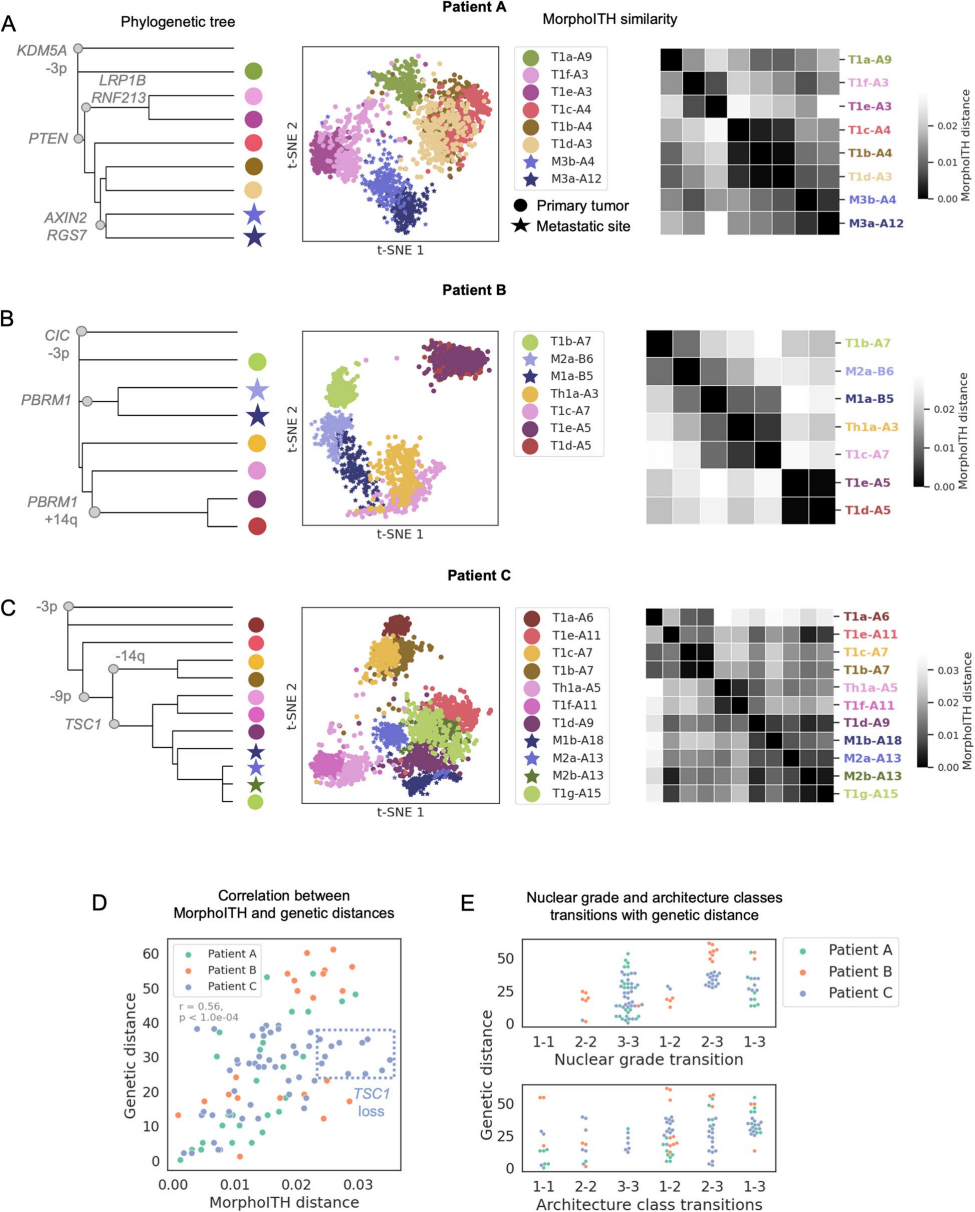
Relationship between morphology and tumor evolution. **A**–**C** We compared phylogenetic trees (“Phylogenetic trees in “Methods”) for three patients (1st column) with their corresponding morphological similarity (visualized using t-SNE in 2nd column and a heatmap in 3rd column with row ordering from the phylogenetic trees) based on the sequencing sample sites and matched local areas within H&E images. Each point in a t-SNE plot is a patch with point color corresponding to its sample site. Primary tumor and metastatic sites are indicated by circles and stars, respectively. In the t-SNE, the samples are named as: T/Mx-block number, with T = primary tumor, and M = metastatic. Each cell in the heatmap represents the pairwise “MorphoITH distance” (cosine distance between mean representative feature vectors of the sampled regions), with lower values indicating greater morphological similarity. **D** Global correlation between genetic distance (*y*-axis) and MorphoITH distance (*x*-axis) for all patients. Mantel’s correlation coefficient *r* = 0.56, with* p*-value < 1e − 4 (999 permutations, two-tailed, stratified by patient). Each point is a pair of samples within a patient that are being compared. **E** Distribution of genetic distances between samples (areas within slides) as a function of clinically used morphologic measures. We check how the genetic distance (*y*-axis) changes when the samples do or do not differ in grade (upper), or in architecture class (lower). Same grade/architecture class transitions are denoted as 1–1, 2–2, and 3–3, while transitions across grade/architecture class include grades/classes 1–2, 2–3, 1–3 (*x*-axis)

To better understand how tumor evolution, which drives genetic ITH, is reflected in morphology, we analyzed multi-regional sequencing data from three patients. For these experiments, each patient had a set of matched whole-exome sequencing and H&E images for morphologically distinct areas within the tumor (WSI-1 cohort in the “Methods” section). Unlike the analyses above, the sample areas were: (a) drawn from different slides to better represent the overall tumor heterogeneity, and (b) selected from subregions of a slide that were morphologically coherent in grade and architecture (Fig. 1C). For each of these patients, we sought to assess the relationship between morphological differences (Fig. 4A–C t-SNE plots and heatmaps, Additional file 1: Fig. S9, S10) and the evolutionary relationships inferred from their phylogenetic trees (Fig. 4A–C, Additional file 1: Fig. S11: trees based on somatic mutations and arm-level copy number alterations; “Phylogenetic trees” in the “Methods” section).

As the different samples were generally easy to distinguish morphologically based on our separability analysis (Additional file 1: Fig. S12), we compared morphological similarity by visualizing individual patch features (Fig. 4A–C t-SNE plots) and by computing cosine distances between the averaged patch features in each area (Fig. 4A–C heatmap plots, Fig. 4D). Patient A displayed concordant evolutionary and morphological patterns with: (a) an early divergence that led to a morphologically distinctive low-grade clone (T1a-A9), (b) a branch containing two clones (T1e-A3, T1f-A3 with mutations in *LRP1B*, *RNF213*) exhibiting similar morphologies, and (c) a clade of 5 samples including two metastases (M3a-A12, M3b-A4 with mutations in *AXIN2* and *RGS7*) that clustered separately based on both genetic and morphological similarity. Patient B showed a comparable pattern with: (a) an early divergence of a low-grade region (T1b-A7) and metastatic branches (M1a-B5, M2a-B6 with *PBRM1 *mutation), and (b) a clade of 4 samples, with a sub-clade (T1d-A5, T1e-A5 with *PBRM1 *mutation and 14q gain) branching further to gain a distinctive morphology. Patient C, while largely reconciling genetic and morphological clustering, presented more genetic complexity among morphologically similar samples. For example, three groups: (i) T1a-A6, (ii) T1b-A7/T1c-A7, and (iii) Th1a-A5/T1f-A11 were clearly separable both morphologically and genetically. However, among the remaining samples, which are all morphologically and genetically similar, T1e-A11 is a genetic outlier that appears to have acquired this morphology via a highly distinctive early branch. Overall, these results demonstrate strong alignment between genetic and morphological similarities across all three patients, albeit with clear outliers.

More globally, we found a strong correlation between genetic similarity and MorphoITH similarity among areas within the same patient (Mantel’s correlation coefficient, *r* = 0.56, *p* < 1e − 4 (stratified by patient); Fig. 4D). Notably, deviations from this trend provided key insights. Rare cases of morphologically similar samples with significant genetic differences were observed, exemplified by T1e-A11 in Patient C (Additional file 1: Fig. S13). Such events are consistent with convergent evolution in our morphological space. Conversely, substantial morphological differences could arise despite divergence in only a small number of genetic events, typically due to the presence of a single highly impactful genetic alteration. For example, 11 outliers with high morphological differences but low genetic variance were all sample pairs that differ in *TSC1* status (Fig. 4D, Additional file 1: Fig. S13). We see a similar trend, although with lower sensitivity, with conventional morphologic descriptors such as grade or architecture. While substantial genetic changes were typically needed for shifts in grade or architecture, regions with identical grades often exhibited significant genetic variability (Fig. 4E). We note that these trends are unlikely to be driven simply by slide-to-slide variations in staining: we consistently observe samples from the same slide being separated, and samples from different slides grouped together, in ways that are consistent with grade and architecture (Additional file 1: Fig. S10). More importantly, staining normalization does not influence our results (Additional file 1: Fig. S14). Taken together, a parsimonious explanation of these observed patterns is that newer clones emerging during tumor evolution typically show incremental changes in morphology, although especially impactful genetic events can lead to larger changes and occasionally distinct genetic trajectories can converge on the same morphological phenotypes. This nuanced relationship between genetic and morphological heterogeneity underscores the utility of MorphoITH in characterizing tumor evolution and phenotypic diversity.

### Impact of encoder choice

Having established that the MorphoITH framework can effectively use features from a task-agnostic, self-supervised encoder to deconvolve ITH from tissue morphology, we investigated how dependent our downstream findings are to the choice of encoder. Specifically, we considered various pathology foundation models which are typically trained using similar self-supervised learning strategies, but on datasets of much larger scale and diversity (though typically not specific to renal cell carcinoma, RCC). As comprehensive downstream testing with all candidate models was impractical, we first benchmarked a selection of these publicly available foundation models on our TMA core retrieval pretext task (Additional file 1: Fig. S15A). From this analysis, we selected UNI [35] (v1), a computationally manageable model that demonstrated strong performance on the pretext task (where UNI and several other foundation models outperformed the version of MorphoITH encoder used in this benchmark, “ViTb/COMBINED”), for detailed comparison in our downstream biological applications.

While direct patch-level similarities between MorphoITH and UNI feature representations showed moderate agreement (Pearson’s *r* = 0.61; Additional file 1: Fig. S15B), the application of the MorphoITH separability framework dramatically increased the concordance (*r* = 0.93; Additional file 1: Fig. S15C, left panel), and the use of control baselines further mitigates systematic differences in separability between encoders (Additional file 1: Fig. S15C, middle and right panels). Consequently, the final biological interpretations remained highly consistent across diverse comparisons (e.g., tumor/non-tumor, grade, architecture, driver gene status; Fig. S16). Similarly, the prognostic effect of the heterogeneity score on TCGA (Additional file 1: Fig. S17A) and the morphology-genetic distance correlation on our multi-region sequencing cohort (Additional file 1: Fig. S17B; Mantel’s correlation coefficient *r* = 0.56 for both MorphoITH and UNI) were largely unaffected. The main exception was morphology-guided subsampling, where our ccRCC-trained encoder performed better for *BAP1*, though relative trends across genes were preserved (Additional file 1: Fig. S18). Overall, these findings demonstrate that MorphoITH’s downstream analytical framework yields robust biological insights that are resilient to variation in the initial self-supervised encoder, as long as the encoder captures relevant morphological structure.

## Discussion

Tumor evolution is arguably the fundamental challenge in oncology. The emergence of subclones in different genetic and epigenetic states makes it challenging to diagnose and treat cancer. Recent multi-region sequencing studies have underscored the inadequacy of profiling single tumor regions in understanding the complexity of most solid tumors [8, 55]. However, the cost associated with multi-region sampling limits its scalability. As tissue morphology reflects the underlying molecular state, we hypothesize that morphological variation in routinely used histopathologic slides, if quantifiable, could serve as a cost-effective proxy for molecular and functional heterogeneity.

Here, we present MorphoITH, a deep learning framework designed to: (a) determine whether areas have similar or distinct morphology, and (b) assess whether these differences relate to spatial variation in other biological properties. We developed MorphoITH in the context of ccRCC, which is known to show extensive intra-tumor heterogeneity [4]. Areas identified as similar by MorphoITH were related; they came from the same TMA core, shared nuclear grade and vascular architecture, or shared other computational image-derived features. By applying MorphoITH to WSIs, we observed abrupt morphological transitions that often correlated with changes in driver mutation status, such as *BAP1* and *SETD2* loss. We also demonstrated how these findings could guide tumor sampling to capture genetic heterogeneity. Furthermore, by quantifying per-slide morphological ITH, we found this measure increased with tumor progression (grade and M stage) and emerged as an independent predictor of progression-free survival beyond grade (being especially prognostic at lower grades). These results, consistent with similar trends observed in genetic-[7] and grade-based [56] heterogeneity, suggest that morphological diversity provides a measure of tumor aggressiveness that complements nuclear grade, which often captures the most advanced phenotype present. Additionally, our analysis of multi-region sequencing in three patients suggests that genetic divergence often leads to commensurate morphological changes, although in some cases, particularly powerful genetic events were associated with disproportionately large morphologic changes. In addition, we observed patterns consistent with convergence on similar morphologies from distinct evolutionary trajectories. Taken together, these findings underscore the complementarity of morphological and genetic analyses to dissect tumor progression.

A key differentiator of MorphoITH is its unbiased nature. While previous studies have used morphology of histopathologic slides to characterize ITH, they have largely focused on specific morphologic phenotypes (e.g., grade in ccRCC [56]) or predefined disease states (e.g., molecular subtypes in breast cancer [57] or pancreatic cancer [58]). In contrast, MorphoITH aims to comprehensively capture morphological changes, serving as a general-purpose framework to explore ITH. To enable MorphoITH to assess morphology without relying on predefined labels, we trained a self-supervised model with hyperparameters [39, 59–62] optimized for this task. We note that this optimization was not critical: our results suggest that the MorphoITH framework can effectively deconvolve morphological ITH utilizing other reasonable task-agnostic representations of morphology, such as from pathology foundation models. To validate the biological relevance of our analyses, we established synthetic baselines to distinguish meaningful correlations from trivial spatial autocorrelations (arising purely from spatially proximal areas being similar [17]), adapting strategies from single-cell and multiplex imaging studies [63, 64]. Using these, we were able to assess how morphology served as a proxy for biologically relevant (genetically informed) ITH, from which prognostically relevant quantitative measures like a per-slide heterogeneity score can be derived.

A particularly striking finding was the presence of sharp morphologic shifts in contiguous areas of ccRCC. Such clear demarcations are characteristic of RCC, in contrast to other cancer types, where the changes are more gradual and thought to be epigenetically driven [65]. Our data suggest that these abrupt transitions represent genetic “clonal” evolution. Specific examples include loss of *BAP1* and *SETD2* (and to a lesser extent *PBRM1*). When these morphologically distinctive regions did not align with *BAP1*/*SETD2* changes, they nonetheless typically behaved like “subclones” in being confined solely to the loss or WT regions.

We note an important distinction in how MorphoITH assesses genetic-morphological links compared to previous approaches from our lab and others predicting genetic events from morphology [14, 15]. MorphoITH tests a-posteriori whether a subclonal genetic event (e.g., *BAP1* loss) is statistically associated with *any* morphological change in a given slide. In contrast, our previous work aimed to predict which regions exhibited *BAP1* loss by identifying global morphological signatures specifically associated with this event across all patients [14]. In other words, MorphoITH evaluates the degree of morphological change associated with a genetic event rather than the morphological distinctiveness of such a change. Multiple genetic events, such as those acting on different levels of the same pathway, may impact morphology similarly and would all be picked up by MorphoITH, but might not be distinguished by a classifier. This may explain why, in contrast to our previous work, we see a stronger link with morphology for *SETD2,* which is associated with higher grade tumors, compared to *PBRM1* whose deficiency is not associated with an increase in grade or decrease in vasculature [11, 66]. Motivated by previous multi-regional sequencing studies in ccRCC, which suggest on average 7 randomly sampled areas are required to capture 75% of driver gene mutations [7, 8], we devised a morphology-guided sequencing strategy. Applied to samples with known subclonal heterogeneity in *BAP1*/*PBRM1*/*SETD2*, we found a significant improvement in the ability to identify this heterogeneity for *BAP1*/*SETD2*, suggesting that a morphology-guided sequencing strategy can indeed be helpful, although improvements may be gene-specific.

Finally, our multi-regional sequencing dataset enabled a preliminary investigation into how tumor evolution is reflected in morphologic heterogeneity. In ccRCC, tumor grade provides only a coarse, monotonic measure of tumor progression. We [13] and others [52] have hypothesized stereotypical trajectories between vascular architectures, and similar trends have been identified in other cancers [67]. Rather than focus on these predefined states, we investigated whether the morphological similarity between regions from the same patient could offer insights into their evolutionary relationships. Our findings suggest that tumor evolution frequently manifests as incremental morphological changes that reflect genetic divergence. However, we also found clear exceptions to this alignment. For example, high-impact genetic events like *TSC1* loss, which is typically associated with mTORC1 activation, were associated with abrupt and drastic changes in morphology. Conversely, we observed instances where genetically distinct evolutionary paths appeared to culminate in strikingly similar morphological phenotypes, a pattern suggestive of convergent phenotypic states. As such, our preliminary findings reveal compelling links between tumor evolution and morphology, highlighting the potential of integrating morphological and genetic analyses to better understand tumor heterogeneity and progression.

There are many avenues to expand upon this work. Firstly, larger studies, such as those leveraging multi-region sequencing datasets like TRACERx Renal [4, 7], are needed to validate our observations. Secondly, future work could explore the relationship between morphology and a broader range of molecular pathways by leveraging spatial transcriptomics. Thirdly, emerging evidence suggests that cellular morphology can actively influence signaling events, expanding upon the concept of structure conforming to function [68]. Thus, MorphoITH may set a foundation for deeper biological insights. Finally, MorphoITH could easily be expanded to other cancer types by utilizing foundation models trained in a pan-cancer context as encoders and adopting techniques to mitigate potential slide-to-slide staining variations [69].

## Conclusions

MorphoITH provides an unbiased, scalable, and versatile framework for studying tumor heterogeneity from histopathologic slides. In the context of ccRCC, our results suggest that morphologically distinct areas are often genetic clones, and the morphological similarity between these areas may reflect their evolutionary relatedness. The ability of morphology to reflect genetic heterogeneity and complement multi-regional studies has practical implications for improving sampling strategies and understanding tumor evolution, offering a new lens to unravel the complexity of cancer biology.

## Supplementary information

Additional file 1.

## Data Availability

The source code is available at the Rajaram Lab public GitHub page https://github.com/Rajaram-Lab/2025-morphoith [70]. H&E images for TCGA KIRC are available from the TCGA GDC portal. TMA Validation and TMA Training are available under the labels TMA1 and TMA2, respectively, at 10.25452/figshare.plus.19324118 [20]. WSI-3 can be accessed at 10.25452/figshare.plus.19310870. [24]. Sequencing data for Patients B and C from WSI-1 (for whom consent has been obtained) is available in the European Genome-phenome Archive (EGA) at https://www.ega-archive.org/studies/EGAS50000001064 [71]. The WSI-1 and WSI-2 image datasets cannot be made publicly available because not all patients provided consent for unrestricted data sharing. The institutional IRB allows use of deidentified data for research purposes, but this does not extend to broad public release. Therefore, we are unable to deposit these datasets in a public repository. We are committed to transparency and will make full effort to provide de-identified data from the WSI-1 and WSI-2 cohorts upon reasonable request, contingent on the establishment of a material transfer agreement between the institutions and in accordance with our IRB guidelines. Requests can be directed to payal.kapur@utsouthwestern.edu. They will typically be reviewed within 2–4 weeks, and data will be shared promptly upon completion of the required agreements.

## References

[1] Lipinski KA, et al. Cancer evolution and the limits of predictability in precision cancer medicine. Trends Cancer. 2016;2:49–63. 10.1016/j.trecan.2015.11.003. 26949746 10.1016/j.trecan.2015.11.003 PMC4756277

[2] Diaz-Colunga J, Diaz-Uriarte R. Conditional prediction of consecutive tumor evolution using cancer progression models: What genotype comes next? PLoS Comput Biol. 2021;17: e1009055. 10.1371/journal.pcbi.1009055. 34932572 10.1371/journal.pcbi.1009055 PMC8730404

[3] Marusyk A, Janiszewska M, Polyak K. Intratumor Heterogeneity: The Rosetta Stone of Therapy Resistance. Cancer Cell. 2020;37:471–84. 10.1016/j.ccell.2020.03.007. 32289271 10.1016/j.ccell.2020.03.007 PMC7181408

[4] Turajlic S, et al. Tracking Cancer Evolution Reveals Constrained Routes to Metastases: TRACERx Renal. Cell. 2018;173(e512), 581–594. 10.1016/j.cell.2018.03.057. 29656895 10.1016/j.cell.2018.03.057 PMC5938365

[5] Gerlinger M, et al. Intratumor heterogeneity and branched evolution revealed by multiregion sequencing. N Engl J Med. 2012;366:883–92. 10.1056/NEJMoa1113205. 22397650 10.1056/NEJMoa1113205 PMC4878653

[6] Pongor LS, Munkácsy G, Vereczkey I, Pete I, Győrffy B. Currently favored sampling practices for tumor sequencing can produce optimal results in the clinical setting. Sci Rep. 2020;10:14403. 10.1038/s41598-020-71382-3. 32873813 10.1038/s41598-020-71382-3 PMC7463012

[7] Turajlic S, et al. Deterministic Evolutionary Trajectories Influence Primary Tumor Growth: TRACERx Renal. Cell 2018;173(e511), 595–610. 10.1016/j.cell.2018.03.043. 29656894 10.1016/j.cell.2018.03.043 PMC5938372

[8] Litchfield K, et al. Representative sequencing: unbiased sampling of solid tumor tissue. Cell Rep. 2020. 10.1016/j.celrep.2020.107550. 32375028 10.1016/j.celrep.2020.107550

[9] Santha P, et al. Morphological heterogeneity in pancreatic cancer reflects structural and functional divergence. Cancers (Basel). 2021. 10.3390/cancers13040895. 33672734 10.3390/cancers13040895 PMC7924365

[10] Aleskandarany MA, et al. Tumour Heterogeneity of Breast Cancer: From Morphology to Personalised Medicine. Pathobiology. 2018;85:23–34. 10.1159/000477851. 29428954 10.1159/000477851

[11] Kapur P, Christie A, Rajaram S, & Brugarolas J. What morphology can teach us about renal cell carcinoma clonal evolution. J Kidney Cancer. 2020. PMC8232548 34178206

[12] Bancroft JD, & Layton C. in Bancroft's theory and practice of histological techniques (Seventh Edition) (Eds S. Kim Suvarna, Christopher Layton, & John D. Bancroft) 173–186 (Churchill Livingstone, 2013).

[13] Cai Q, et al. Ontological analyses reveal clinically-significant clear cell renal cell carcinoma subtypes with convergent evolutionary trajectories into an aggressive type. EBioMedicine. 2020;51: 102526. 10.1016/j.ebiom.2019.10.052. 31859241 10.1016/j.ebiom.2019.10.052 PMC7000318

[14] Acosta PH, et al. Intratumoral resolution of driver gene mutation heterogeneity in renal cancer using deep learning. Cancer Res. 2022;82:2792–806. 10.1158/0008-5472.CAN-21-2318. 35654752 10.1158/0008-5472.CAN-21-2318 PMC9373732

[15] Bychkov D, et al. Deep learning identifies morphological features in breast cancer predictive of cancer ERBB2 status and trastuzumab treatment efficacy. Sci Rep. 2021;11:4037. 10.1038/s41598-021-83102-6. 33597560 10.1038/s41598-021-83102-6 PMC7890057

[16] Gerlinger M, et al. Genomic architecture and evolution of clear cell renal cell carcinomas defined by multiregion sequencing. Nat Genet. 2014;46:225–33. 10.1038/ng.2891. 24487277 10.1038/ng.2891 PMC4636053

[17] Rajaram S, et al. Sampling strategies to capture single-cell heterogeneity. Nat Methods. 2017;14:967–70. 10.1038/nmeth.4427. 28869755 10.1038/nmeth.4427 PMC5658002

[18] Gayed BA, et al. Prognostic role of cell cycle and proliferative biomarkers in patients with clear cell renal cell carcinoma. J Urol. 2013;190:1662–7. 10.1016/j.juro.2013.06.037. 23792148 10.1016/j.juro.2013.06.037

[19] Darwish OM, et al. Cumulative number of altered biomarkers in mammalian target of rapamycin pathway is an independent predictor of outcome in patients with clear cell renal cell carcinoma. Urology. 2013;81:581–6. 10.1016/j.urology.2012.11.030. 23290145 10.1016/j.urology.2012.11.030

[20] Rajaram S, et al. Testing Cohort Slide Images for "Intratumoral resolution of driver gene mutation heterogeneity in renal cancer using deep learning", Figshare+. 202110.25452/figshare.plus.19324118

[21] Kapur P, et al. Effects on survival of BAP1 and PBRM1 mutations in sporadic clear-cell renal-cell carcinoma: a retrospective analysis with independent validation. Lancet Oncol. 2013;14:159–67. 10.1016/S1470-2045(12)70584-3. 23333114 10.1016/S1470-2045(12)70584-3 PMC4674067

[22] Jasti J, et al. Histopathology based AI model predicts anti-angiogenic therapy response in renal cancer clinical trial. Nat Commun. 2025;16:2610. 10.1038/s41467-025-57717-6. 40097393 10.1038/s41467-025-57717-6 PMC11914575

[23] Joseph RW, et al. Clear cell renal cell carcinoma subtypes identified by BAP1 and PBRM1 expression. J Urol. 2016;195:180–7. 10.1016/j.juro.2015.07.113. 26300218 10.1016/j.juro.2015.07.113 PMC5221690

[24] Rajaram S, et al. Training cohort slide images for "intratumoral resolution of driver gene mutation heterogeneity in renal cancer using deep learning", Figshare+. 202210.25452/figshare.plus.19310870.

[25] Ricketts CJ, et al. The Cancer Genome Atlas Comprehensive Molecular Characterization of Renal Cell Carcinoma. Cell Rep. 2018;23(e315), 313–326. 10.1016/j.celrep.2018.03.075. 29617669 10.1016/j.celrep.2018.03.075 PMC6075733

[26] Grill J, et al. Bootstrap your own latent a new approach to self-supervised learning. arXiv 2020. 10.48550/arXiv.2006.07733.

[27] Chen, X. & He, K. Exploring simple siamese representation learning. 2020arXiv:2011.10566. 10.48550/arXiv.2011.10566.

[28] Wang P. Bootstrap your own latent (BYOL), in Pytorch, Github repository, 2023https://github.com/lucidrains/byol-pytorch.

[29] Musgrave K, Belongie SJ, & Lim S. Pytorch metric learning. arXiv 2020. arXiv:2008.09164v1

[30] Chen X, Fan H, Girshick R & He K. Improved baselines with momentum contrastive learning. arXiv 2020. 10.48550/arXiv.2003.04297

[31] Dosovitskiy A, et al. An image is worth 16x16 words: transformers for image recognition at scale. arXiv 2020. 10.48550/arXiv.2010.11929

[32] He K, Zhang X, Ren S, & Sun J. Deep residual learning for image recognition. arXiv 2015. 10.48550/arXiv.1512.03385.

[33] Deng J, et al. in 2009 IEEE Conference on computer vision and pattern recognition. Edition edn (2009 Published).

[34] Yan C. Augmentation-pytorch-transforms, GitHub repository. 2021https://github.com/gatsby2016/Augmentation-PyTorch-Transforms.

[35] Chen RJ, et al. Towards a general-purpose foundation model for computational pathology. Nat Med. 2024;30:850–62. 10.1038/s41591-024-02857-3. 38504018 10.1038/s41591-024-02857-3 PMC11403354

[36] Vorontsov E, et al. A foundation model for clinical-grade computational pathology and rare cancers detection. Nat Med. 2024;30:2924–35. 10.1038/s41591-024-03141-0. 39039250 10.1038/s41591-024-03141-0 PMC11485232

[37] Xu H, et al. A whole-slide foundation model for digital pathology from real-world data. Nature. 2024;630:181–8. 10.1038/s41586-024-07441-w. 38778098 10.1038/s41586-024-07441-w PMC11153137

[38] Lu MY, et al. A visual-language foundation model for computational pathology. Nat Med. 2024;30:863–74. 10.1038/s41591-024-02856-4. 38504017 10.1038/s41591-024-02856-4 PMC11384335

[39] Wang X, et al. RetCCL: clustering-guided contrastive learning for whole-slide image retrieval. Med Image Anal. 2023;83:102645. 10.1016/j.media.2022.102645. 36270093 10.1016/j.media.2022.102645

[40] Weng Z, et al. Grandqc: a comprehensive solution to quality control problem in digital pathology. Nat Commun. 2024;15:10685. 10.1038/s41467-024-54769-y. 39681557 10.1038/s41467-024-54769-y PMC11649692

[41] Weigert M, Schmidt U, Haase R, Sugawara K, & Myers G. Star-convex Polyhedra for 3D object detection and segmentation in microscopy. arXiv 2019. 10.48550/arXiv.1908.03636

[42] Schmidt U, Weigert M, Broaddus C, & Myers G. Edition edn (MICCAI 2018, 2018 Published). 10.48550/arXiv.1806.03535.

[43] Raulerson CK, et al. SCHOOL: Software for clinical health in oncology for omics laboratories. J Pathol Inform. 2022;13:100163. 10.4103/jpi.jpi_20_21. 35136669 10.4103/jpi.jpi_20_21 PMC8794024

[44] Cibulskis K, et al. Sensitive detection of somatic point mutations in impure and heterogeneous cancer samples. Nat Biotechnol. 2013;31:213–9. 10.1038/nbt.2514. 23396013 10.1038/nbt.2514 PMC3833702

[45] Garrison EP, & Marth GT. Haplotype-based variant detection from short-read sequencing. arXiv: Genomics arXiv:1207.3907 2012. http://arxiv.org/abs/1207.3907.

[46] Kim S, et al. Strelka2: fast and accurate calling of germline and somatic variants. Nat Methods. 2018;15:591–4. 10.1038/s41592-018-0051-x. 30013048 10.1038/s41592-018-0051-x

[47] Shen R, Seshan VE. FACETS: allele-specific copy number and clonal heterogeneity analysis tool for high-throughput DNA sequencing. Nucleic Acids Res. 2016;44: e131. 10.1093/nar/gkw520. 27270079 10.1093/nar/gkw520 PMC5027494

[48] Sondka Z, et al. COSMIC: a curated database of somatic variants and clinical data for cancer. Nucleic Acids Res. 2023;52:D1210–7. 10.1093/nar/gkad986. 10.1093/nar/gkad986 PMC10767972 38183204

[49] Macenko M, et al. in 2009 IEEE International symposium on biomedical imaging: From Nano to Macro. Edition edn 1107–1110 (2009 Published). 10.1109/ISBI.2009.5193250

[50] Vahadane A, et al. Structure-Preserving Color Normalization and Sparse Stain Separation for Histological Images. IEEE Trans Med Imaging. 2016;35:1962–71. 10.1109/TMI.2016.2529665. 27164577 10.1109/TMI.2016.2529665

[51] Mantel N. The detection of disease clustering and a generalized regression approach. Cancer Res. 1967;27:209–20. 6018555

[52] Sirohi D, et al. Histologic Growth Patterns in Clear Cell Renal Cell Carcinoma Stratify Patients into Survival Risk Groups. Clin Genitourin Cancer. 2022;20:e233–43. 10.1016/j.clgc.2022.01.005. 35125301 10.1016/j.clgc.2022.01.005

[53] Ohe C, et al. Development and validation of a vascularity-based architectural classification for clear cell renal cell carcinoma: correlation with conventional pathological prognostic factors, gene expression patterns, and clinical outcomes. Mod Pathol. 2022;35:816–24. 10.1038/s41379-021-00982-9. 34848832 10.1038/s41379-021-00982-9

[54] Moch H. The WHO/ISUP grading system for renal carcinoma. Pathologe. 2016;37:355–60. 10.1007/s00292-016-0171-y. 27271258 10.1007/s00292-016-0171-y

[55] Kader T, Zethoven M, Gorringe KL. Evaluating statistical approaches to define clonal origin of tumours using bulk DNA sequencing: context is everything. Genome Biol. 2022;23:43. 10.1186/s13059-022-02600-6. 35109903 10.1186/s13059-022-02600-6 PMC8809045

[56] Nyman J, et al. Spatially aware deep learning reveals tumor heterogeneity patterns that encode distinct kidney cancer states. Cell Rep Med. 2023;4:101189. 10.1016/j.xcrm.2023.101189. 37729872 10.1016/j.xcrm.2023.101189 PMC10518628

[57] Zormpas-Petridis K, et al. SuperHistopath: A Deep Learning Pipeline for Mapping Tumor Heterogeneity on Low-Resolution Whole-Slide Digital Histopathology Images. Front Oncol. 2020;10: 586292. 10.3389/fonc.2020.586292. 33552964 10.3389/fonc.2020.586292 PMC7855703

[58] Saillard C, et al. Pacpaint: a histology-based deep learning model uncovers the extensive intratumor molecular heterogeneity of pancreatic adenocarcinoma. Nat Commun. 2023;14:3459. 10.1038/s41467-023-39026-y. 37311751 10.1038/s41467-023-39026-y PMC10264377

[59] Ciga O, Xu T, Martel AL. Self supervised contrastive learning for digital histopathology. machine learning with applications. 2022. 10.1016/j.mlwa.2021.100198.

[60] DiPalma J, Torresani L, Hassanpour S. Histoperm: a permutation-based view generation approach for improving histopathologic feature representation learning. J Pathol Inform. 2023;14:100320. 10.1016/j.jpi.2023.100320. 37457594 10.1016/j.jpi.2023.100320 PMC10339175

[61] Schirris Y, Gavves E, Nederlof I, Horlings HM, Teuwen J. Deepsmile: Contrastive self-supervised pre-training benefits MSI and HRD classification directly from H&E whole-slide images in colorectal and breast cancer. Med Image Anal. 2022;79:102464. 10.1016/j.media.2022.102464. 35596966 10.1016/j.media.2022.102464

[62] Komura D, et al. Universal encoding of pan-cancer histology by deep texture representations. Cell Rep. 2022;38:110424. 10.1016/j.celrep.2022.110424. 35235802 10.1016/j.celrep.2022.110424

[63] Bost P, Schulz D, Engler S, Wasserfall C, Bodenmiller B. Optimizing multiplexed imaging experimental design through tissue spatial segregation estimation. Nat Methods. 2023;20:418–23. 10.1038/s41592-022-01692-z. 36585456 10.1038/s41592-022-01692-z PMC9998266

[64] Baker EAG, Schapiro D, Dumitrascu B, Vickovic S, Regev A. In silico tissue generation and power analysis for spatial omics. Nat Methods. 2023;20:424–31. 10.1038/s41592-023-01766-6. 36864197 10.1038/s41592-023-01766-6 PMC9998272

[65] Lin JR, et al. Multiplexed 3D atlas of state transitions and immune interaction in colorectal cancer. Cell. 2023;e319, 363–381. 10.1016/j.cell.2022.12.028 10.1016/j.cell.2022.12.028 PMC10019067 36669472

[66] Ho TH, et al. Loss of histone H3 lysine 36 trimethylation is associated with an increased risk of renal cell carcinoma-specific death. Mod Pathol. 2016;29:34–42. 10.1038/modpathol.2015.123. 26516698 10.1038/modpathol.2015.123 PMC4697879

[67] Tavernari D, et al. Nongenetic evolution drives lung adenocarcinoma spatial heterogeneity and progression. Cancer Discov. 2021;11:1490–507. 10.1158/2159-8290.Cd-20-1274. 33563664 10.1158/2159-8290.CD-20-1274

[68] Weems AD, et al. Blebs promote cell survival by assembling oncogenic signalling hubs. Nature. 2023;615:517–25. 10.1038/s41586-023-05758-6. 36859545 10.1038/s41586-023-05758-6 PMC10881276

[69] Shen Y, Luo Y, Shen D, & Ke J. in Medical image computing and computer assisted intervention – MICCAI 2022 Edition edn 212–221 (2022 Published).

[70] Nielsen AW, et al. MorphoITH, GitHub repository. 2025. https://github.com/Rajaram-Lab/2025-morphoith.

[71] MorphoI TH. A Framework for Deconvolving Intra-Tumor Heterogeneity Using Tissue Morphology, European Genomephenome archive. 2025:EGAD50000001550. 10.1186/s13073-025-01504-x PMC12447597 40968388

