# Supplementary material for: MorphoITH: a framework for deconvolving intra-tumor heterogeneity using tissue morphology
Source: Genome Med. 2025 Sep 19;17:101. doi: 10.1186/s13073-025-01504-x (PMC12447597; doi:10.1186/s13073-025-01504-x)

Table S1. Summary of datasets used in this study, their characteristics, and primary applications

Dataset	Number of slides	Number of patients	Analyses	Patch extraction	Slides magnification
TMA Training	10 (785 cores, ~1mm diameter)	421	1. Model development.<br>2. Model comparison (approximately 2/3 of the dataset used for training, and 1/3 for testing).	Random selection of 100-200 patches per core;<br>153,968 patches total.	40X
TMA Validation	3 (183 cores, ~1mm diameter)	95	Model validation (same core and same nuclear grade retrieval task).	Random selection of 100-200 patches per core;<br>32,780 patches total.	20X
WSI-1	41	17<br>3 of them have matching DNA sequencing.	1. At least two different nuclear grades sites (16 WSIs) and two different vascular architectures sites (26 WSIs) per slide annotated by a pathologist.<br>2. Multi-region sequencing performed for 3 patients (18 WSIs). Number of samples per patient: Patient A=8 (6 primary sites, 2 small intestine metastatic site), Patient B=7 (5 primary sites, 2 pancreas metastatic site), Patient C=11 (8 primary sites, 2 adrenal metastatic sites (M2), 1 lymph node metastatic site (M1)). Average size of the sample site: 6.6mm^2.	1. Tessellation of WSIs with stride equal to 100 px.<br>2. Random selection of maximum 250 patches per region marked for multi-region sequencing; 6,500 patches total.	40X and 20X
WSI-2	10	10	1. At least two different nuclear grades sites (4 WSIs) and at two different vascular architectures sites (7 WSIs) per slide annotated by a pathologist.<br>2. Handcrafted features analyzed from tumor areas of whole slide images (10 WSIs).	Tessellation of WSIs with stride equal to 100 px.	20X
WSI-3	1268<br>Of 1362 total WSIs from this cohort, 1268 were used in this study after filtering out slides that have <100px of tumor areas after tessellation.	1268	1. All WSIs with known driver gene status of wild-type (WT) or loss, as assessed by immunochemistry (IHC) assays done on serial sections, utilized as an input for heterogeneity score calculations.<br>2. Subset of cases that exhibited areas with both loss and WT driver gene status within the same slide (18 BAP1, 9 SETD2, and 9 PBRM1) were additionally annotated for their localized (focal) loss.	Tessellation of WSIs with stride equal to 100 px.	20X
TCGA KIRC	444<br>Of 519 total WSI from this cohort, 444 were used in this study after filtering out slides that have <100px of tumor areas after tessellation, and after image quality control as described previously. 348 patients were used after additional filtering for patients that have clinical data for nuclear grade, survival time, and mStage.	444	1. Separation of tumor from non-tumor areas (444 patients)<br>2. Heterogeneity and survival analysis (348 patients).	Tessellation of WSIs with stride equal to 100 px.	40X and 20X

Fig. S1. A) Comparison of training strategies for MorphoITH development. Description of different approaches that were compared using the retrieval task on the held-out portion of the TMA Training cohort. The approaches differ in model backbones (ViTb, ResNet50), training strategies (BYOL, Triplet, MoCov2), and input types ("combined", "same", "close"). B) Comparison of the pretext retrieval task, in which we calculate the fraction of patches (y-axis) for which the N-th nearest neighbors (x-axis) were from the same TMA core for different strategies. C) Retrieval task results (y-axis, fraction of patches captured from the same TMA core) at the 10th nearest neighbor for different strategies (x-axis).

A

Training strategy	Backbones	Input (positive pair definition) [1]
BYOL	ViTb	Combined<br>Close<br>Same
	ResNet50	
Triplet	ViTb	Combined<br>Close<br>Same
	ResNet50	
MoCov2 [2]	ResNet50	Close<br>Same

[1] MorphoITH encoder: training and selection in Methods
Same = same patch augmented
Close = close spatially patches augmented
Combined = combination of the above

[2] Pipeline used as provided by the authors, without switching the encoders or adding custom "combined" DataLoader

B

C

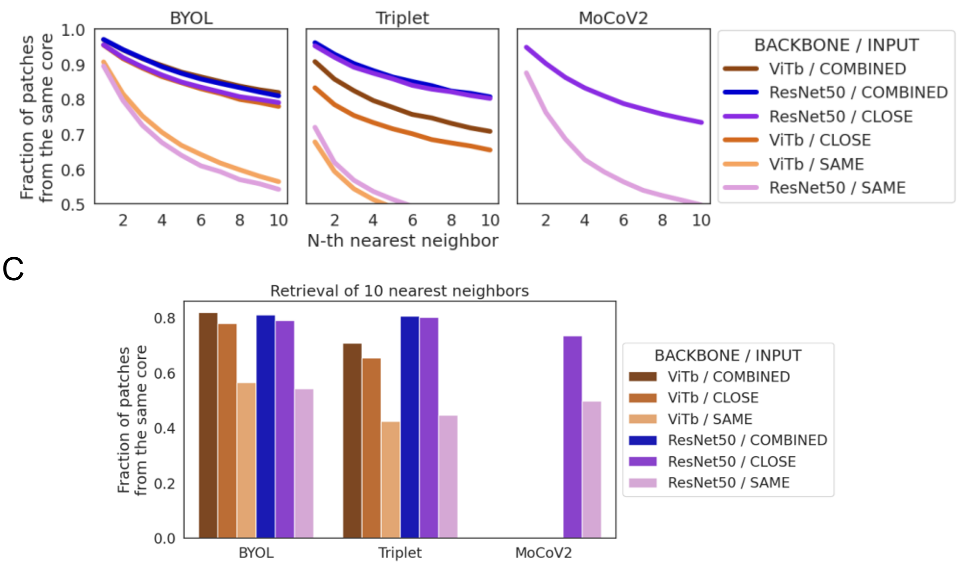

Fig. S2. MorphoITH-based similarity captures biologically relevant aspects of morphology. For each patch in the independent "TMA Validation" cohort, we ranked all other patches based on their similarity. A) An example of one patch and its 9 closest neighbors (G=grade, N=nearest neighbor rank). B-C) MorphoITH similarity across TMA cores and grades. B) Stacked plot showing the fraction of patches (y-axis) for which the N-th nearest neighbor (x-axis) was from the same TMA core (orange), had the same nuclear grade (but from a different core, blue), or differed in both core and grade (grey). Fractions are stacked and sum to 1. C) Box-plot showing the average nearest-neighbor ranks of patches from each of these classes. D) Global view of MorphoITH's feature space: t-SNE based dimensional reduction was used to visualize the similarity between the MorphoITH vectors for a random subset of 10,000 patches. Each point represents a patch from a TMA core. Points are colored based on the core they were derived from, with patches from one example core highlighted in red and the corresponding patch image depicted on the side. Points' positions are the same as in Fig. 1A, except there patches are colored by grade.

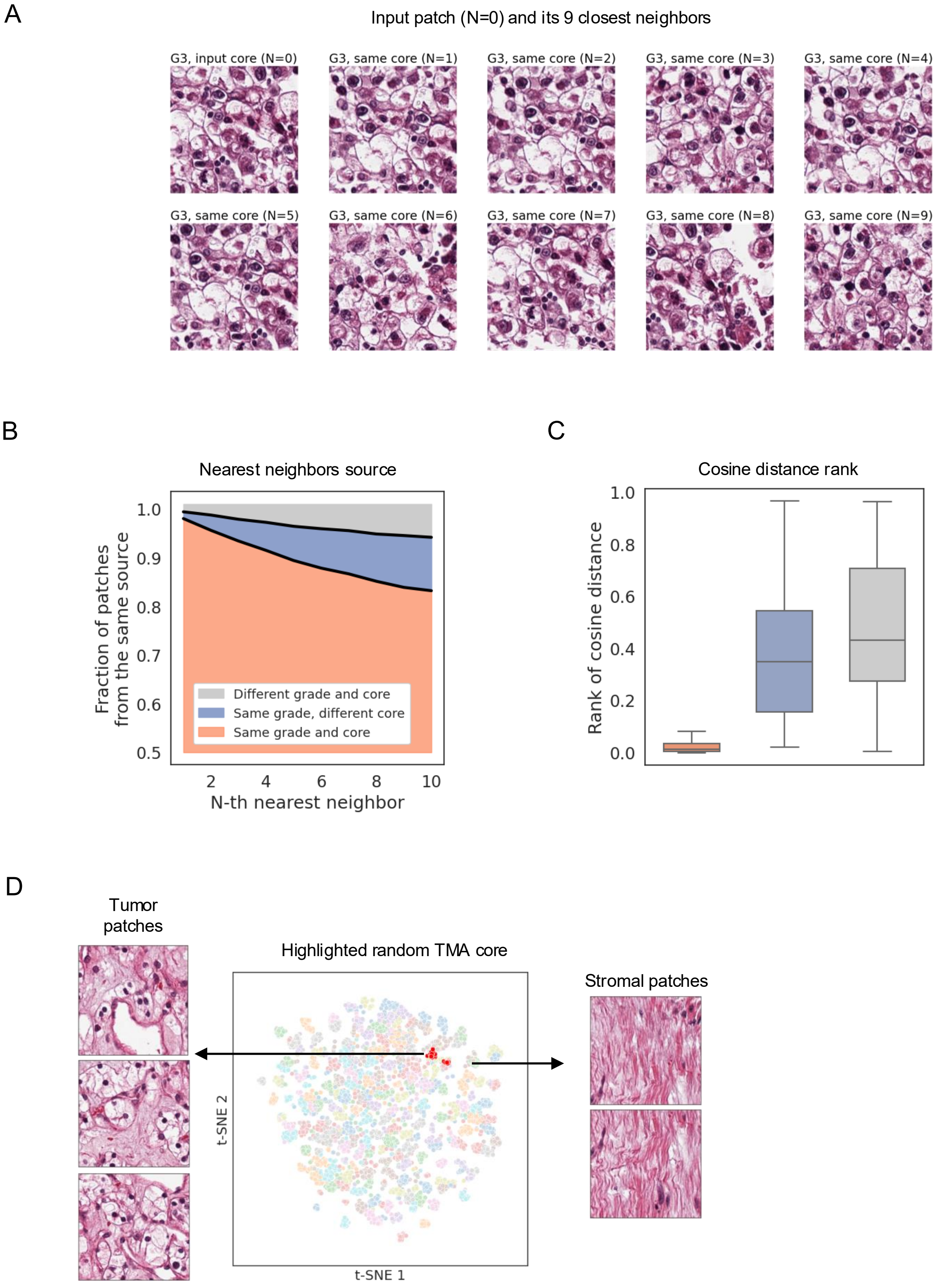

Fig. S3. MorphoITH visualization of tissue-type deconvolution on TCGA KIRC dataset. A-B) Two examples comparing whole slide MorphoITH profiles to different tissue region types. Tissue type ground truth (1st rows) was obtained using an in-house deep learning "Region Classifier". MorphoITH features were extracted across patches in the slide (2nd rows) and visualized with pseudo-colors, where similar colors indicate similarity in morphology. Separation of tumor vs non-tumor areas was performed on MorphoITH-derived feature vectors using an SVM algorithm, which we visualize using t-SNE on 1,000 random patches per slide and color based on true tissue class and MorphoITH pseudo color. Note: in example B there is significant heterogeneity within the tumor class.

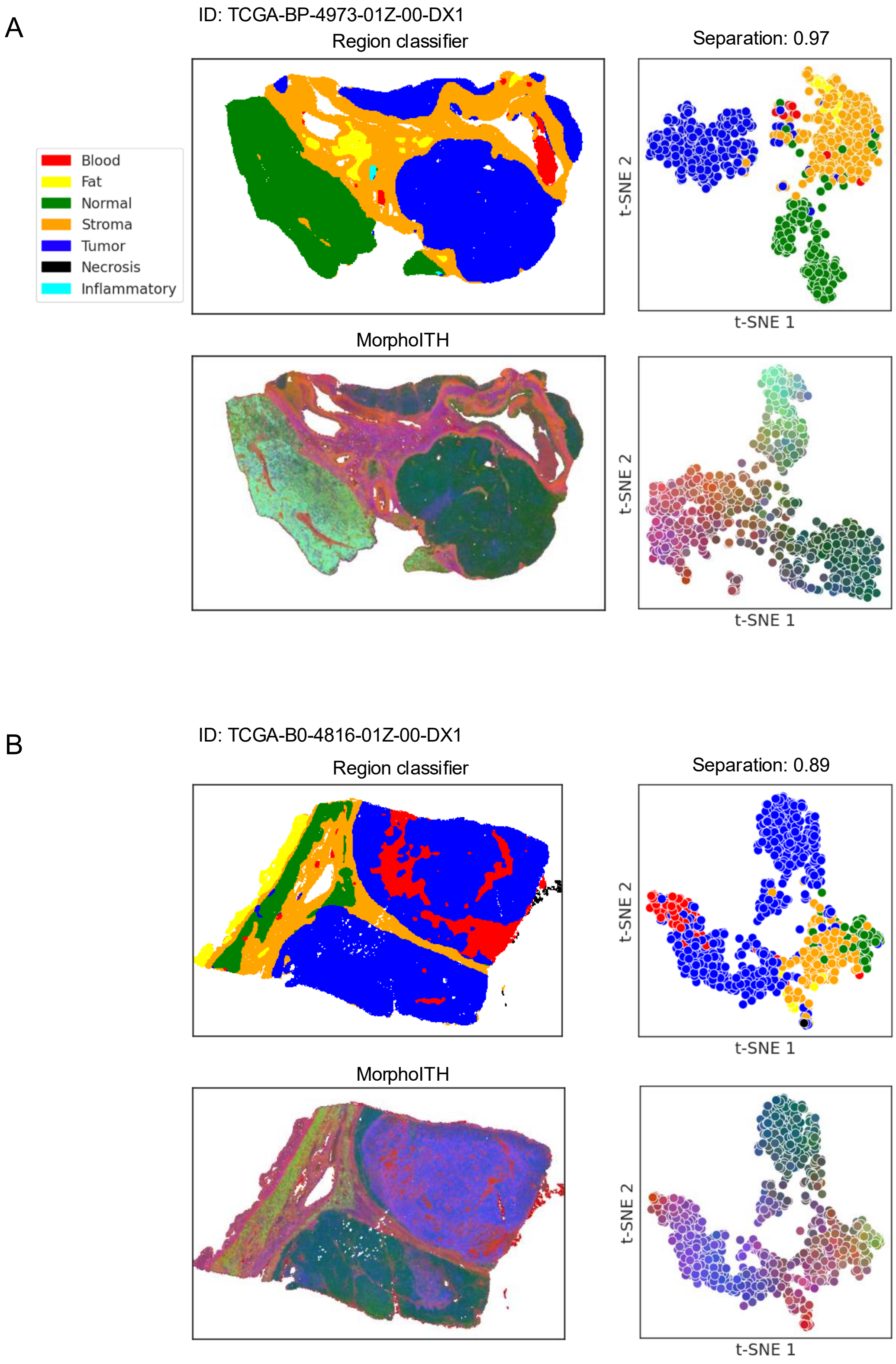

Fig. S4. Examples of A) full, B) partial, and C) no separation of tumor vs non-tumor areas in TCGA KIRC based on MorphoITH-derived feature vectors. We show input H&E images (1st column) with their MorphoITH output in form of pseudo-colors that indicate morphological similarity (2nd column), as well as "Region Classifier" output and its simplification of tumor vs non-tumor tissue classes (3rd and 4th columns, respectively). In A), we provide legend to read region classifier output (3rd column) and its categorization into tumor vs non-tumor regions (4th column).

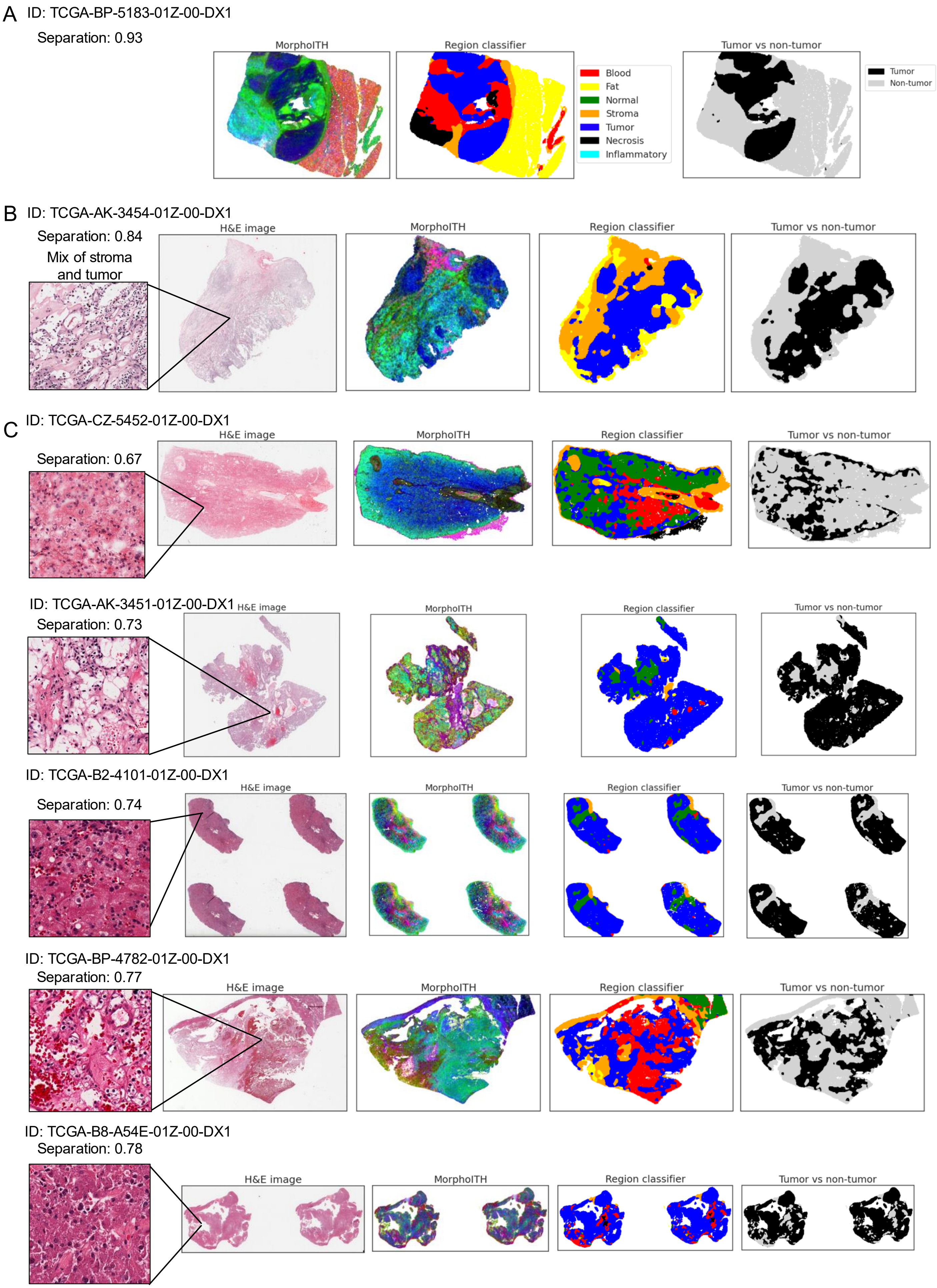

Fig. S5. Distinct classes of vascular architectures and nuclear grade show separation based on MorphoITH similarity measure. Separation measure (y-axis) of: A) vascular architectures and B) nuclear grades. X-axis shows pairs of architectures/grades: each point in a plot corresponds to one pair of within-slide regions that were compared. Corresponding contiguity baselines and negative controls are indicated in grey. Notable exceptions to strong separation include comparisons between Grade 1 vs. Grade 2, and between "small nest" vs. "large nest" architectures, reflecting their close morphological similarities. In A, different point shapes indicate whether there was a nuclear grade change between the pair that already differs in architecture.

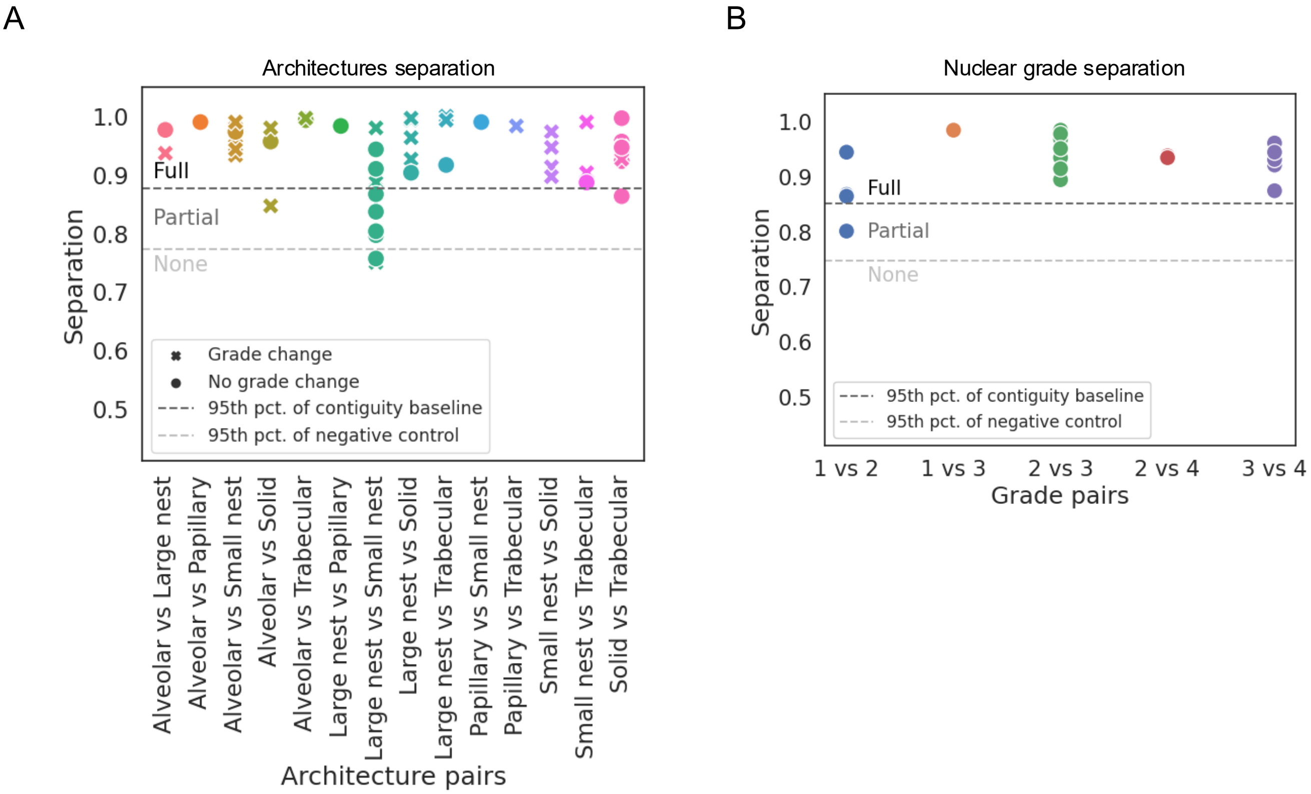

Fig. S6. MorphoITH encompasses a broad range of morphological descriptors. A) Examples of different morphological descriptors heatmaps (nucleus size, vasculature density, eosin intensity) as compared to our measure of similarity, with output visualization pseudo-colors indicating morphological similarity. B) While MorphoITH can recognize three distinct areas A, B, and C, the individual descriptors might be undistinguishable between them (reported by Wilcoxon rank-sum test**)**. The sampled areas are indicated in the MorphoITH map with "x", and span 25 adjacent patches, with each dot in the box-plots describing mean feature value within a single patch. C) Relationships between most similar patches selected based on different descriptors. For the same slide as shown in A), we calculated 100 pairs of patches that were most similar based on the "selection features" (y-axis; morphological similarity, vasculature density, eosin intensity, random), and calculated their scaled rank (x-axis, most similar/dissimilar pair have rank 0/100, respectively) based on difference in "comparison feature" (nucleus size). "Random" row describes difference in nucleus size if patches were chosen randomly. D) The same analysis as in C), averaged cross all slides and calculated using all combinations of selection features (y-axis) and comparison features (x-axis, as opposed to just nucleus size in C). While in general the ranks of the nearest neighbors for one feature were no better than a random ranking relative to a different feature, the MorphoITH based ranking consistently succeeded in capturing points deemed similar by all other descriptors.

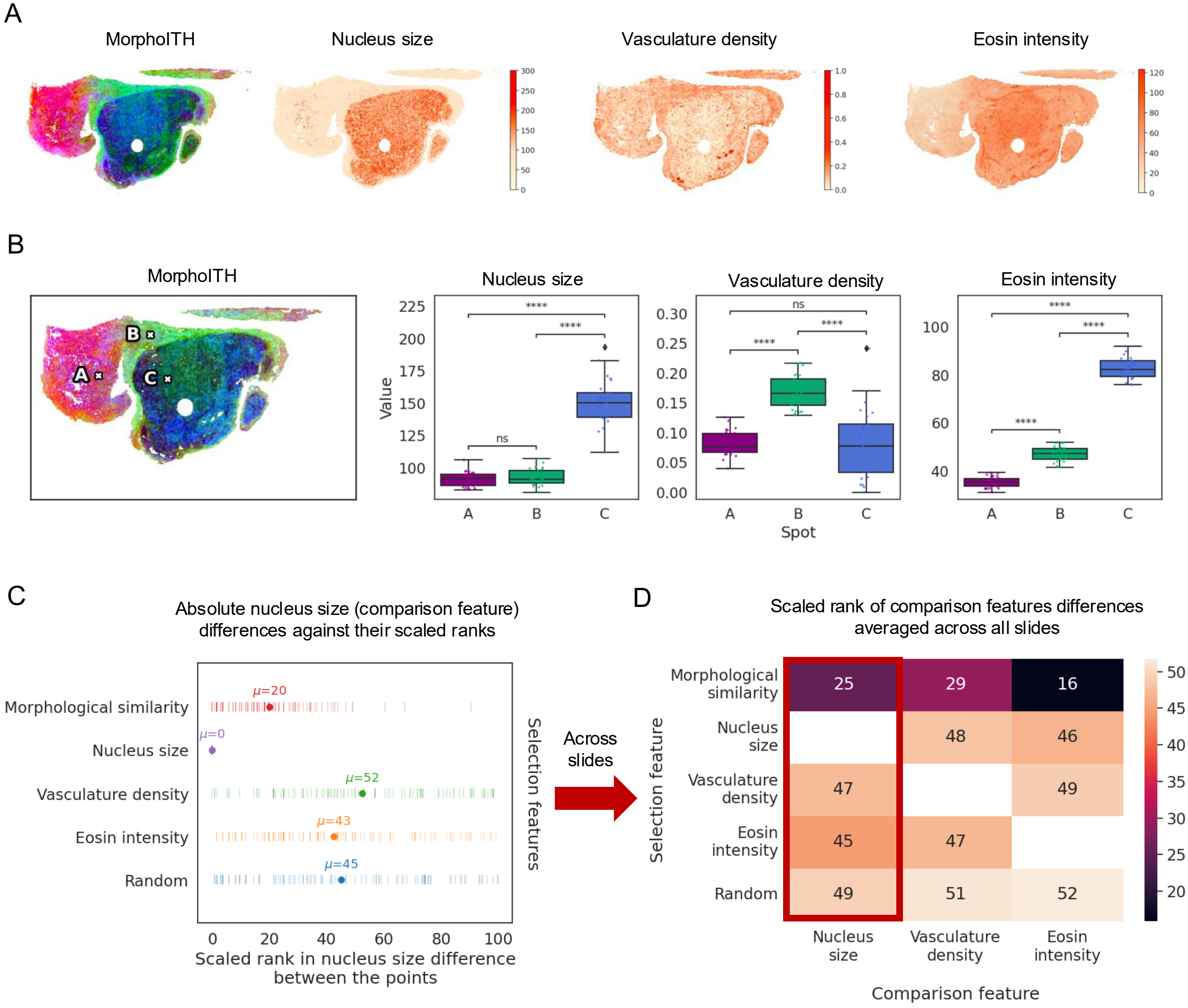

Fig. S7. A) Examples of a) full, b) partial, and c) no separation between ground truth annotations for chosen examples of driver mutations. Left column: ground truth based on pathologist's annotations. Right column: visualization of MorphoITH output with pseudo-colors which indicate similarity in morphology. B) Overlap Calculation: 1st column: H&E image. 2nd column: MorphoITH pseudo-color visualization within tumor regions. 3rd column: Resulting N=10 spatially constrained morphological clusters. 4th column: Visualization of each cluster's fractional overlap with the "loss" region. 5th column: Pathologist-annotated ground truth WT and driver gene loss regions. C) Percentage of morphological clusters spanning both WT and loss regions (defined as 0.2 < overlap < 0.8 with loss region) for different driver genes, for number of clusters = 5, 10, 15, 20. We compared the overlap of annotated loss areas (ground truth, purple) with corresponding controls (grey). Each bar has number of split clusters indicated on top of it (N). D) Distribution of the fractional loss area per slide for WSI-3 cases heterogeneous for *BAP1*, *SETD2*, and *PBRM1*. Each horizontal line within a column represents one slide, indicating the proportion of the tumor area that corresponds to driver gene loss.

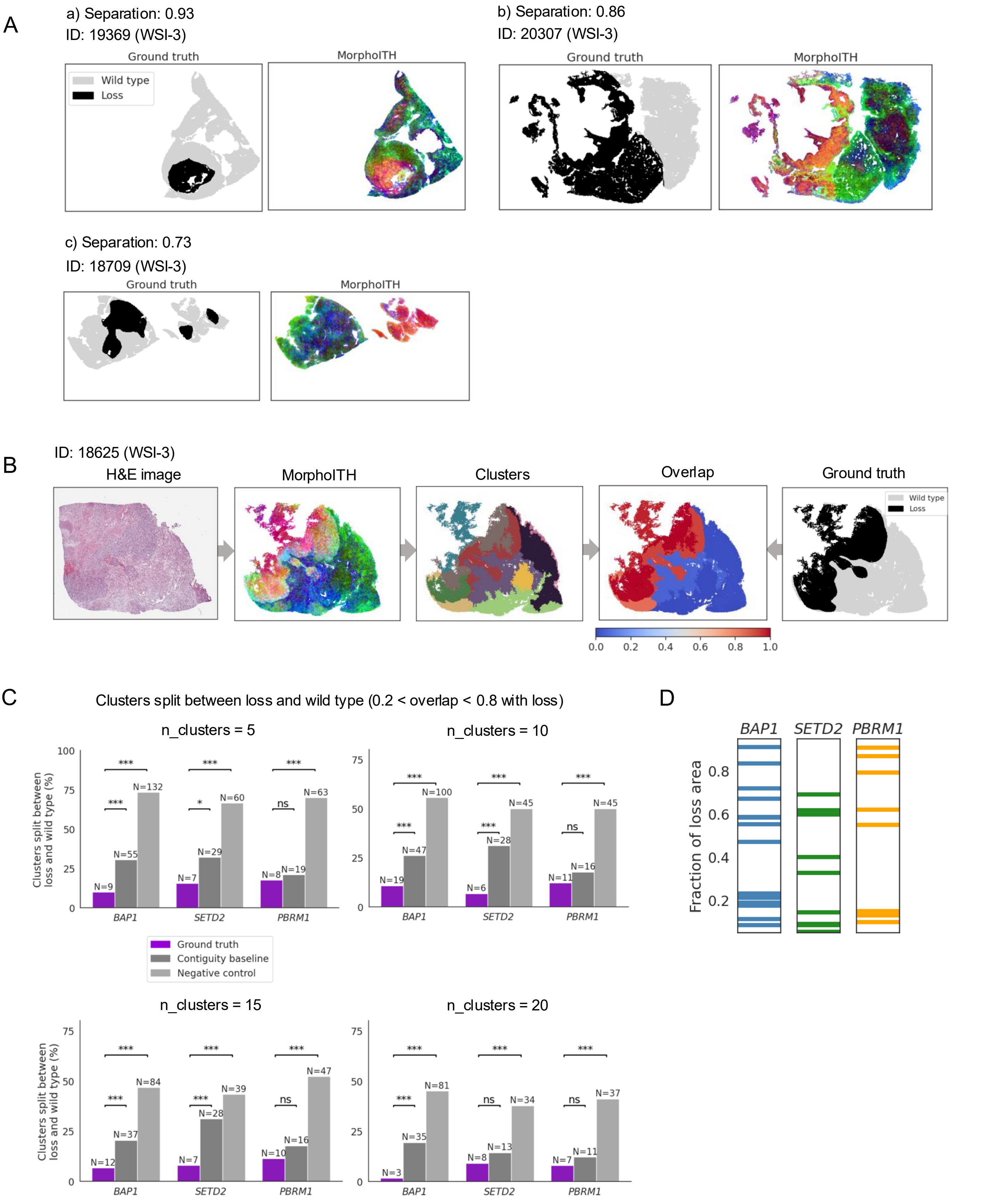

Fig. S8. Single-slide MorphoITH heterogeneity score: association with genetic ITH and clinical variables. A) Distribution of single slide heterogeneity scores (x-axis) for WSIs (green) from the WSI-3 dataset. For comparison, distributions of a TMA core-level heterogeneity metric (median intra-core pairwise MorphoITH patch distances; blue) from the TMA Validation dataset are shown. Left: example TMA cores with low and high heterogeneity. B) Per-slide MorphoITH heterogeneity scores (y-axis) in the WSI-3 dataset, comparing focal cases (intra-slide genetic ITH for *BAP1*, *PBRM1*, or *SETD2*) to non-focal (homogenous WT/loss) slides for each respective gene (reported by Wilcoxon rank-sum test). C) Heterogeneity scores (y-axis) by nuclear grade (x-axis) in TCGA KIRC. High grade = grades 3, 4. Low grade = grade 1, 2. Slides with higher nuclear grades are significantly more heterogeneous than those with lower grade (reported by Wilcoxon rank-sum test). D) Heterogeneity scores (y-axis) by mStage (x-axis) in TCGA KIRC. Even when limited to high grade only (right side), heterogeneity scores are significantly correlated with mStage (reported by Wilcoxon rank-sum test). E, F) Kaplan-Meier Progression-Free Survival analysis in TCGA KIRC stratified by median heterogeneity score calculated for: E) all patients but with different number of clusters N = 5, 15, 20, F) N = 10 but also split by grade (left) and only for low grade (right) where the heterogeneity score has higher predictive power. G) Heterogeneity scores for each slide of Patients A-C from cohort WSI-1. ANOVA test performed to check for variance in heterogeneity score explained by patient effect.

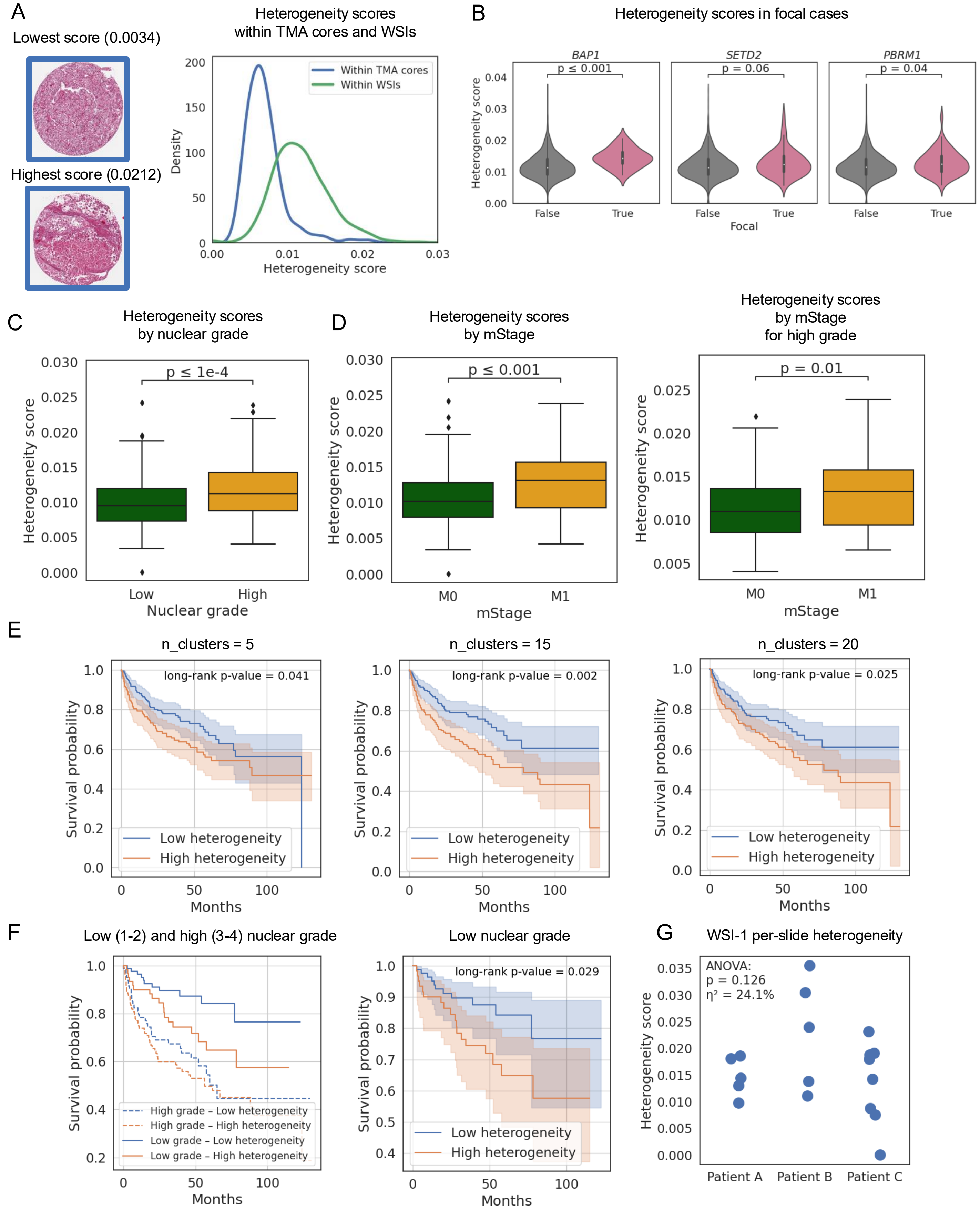

Fig. S9. A-C) t-SNE plots of morphological similarity with overlayed patches from which the MorphoITH feature space was derived.

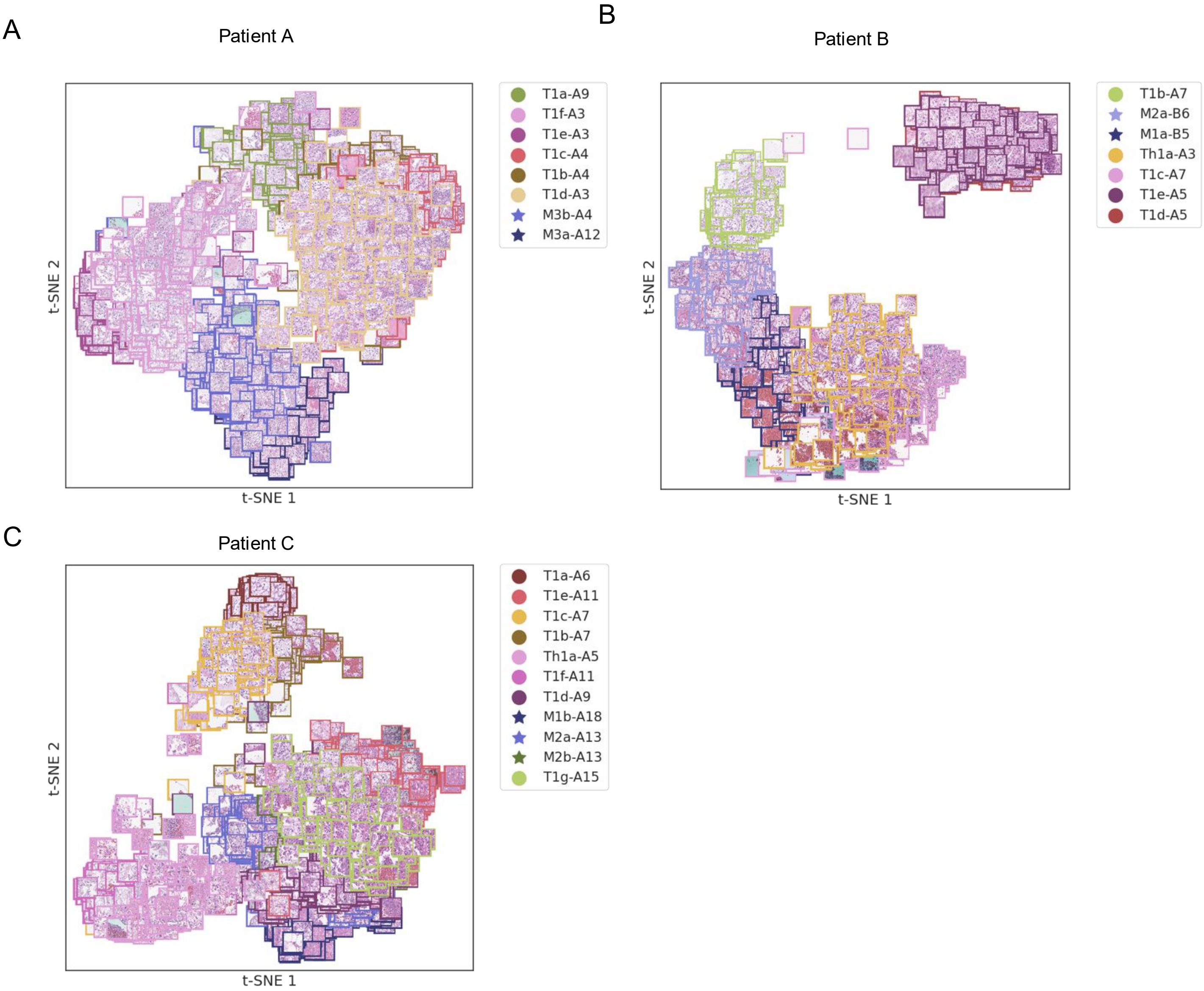

Fig. S10. A-C) t-SNE plots of morphological similarity (each point represents one patch) and phylogenetic trees for patients A-C with overlayed nuclear grade, vascular architectures annotations, as well as H&E slide of origin.

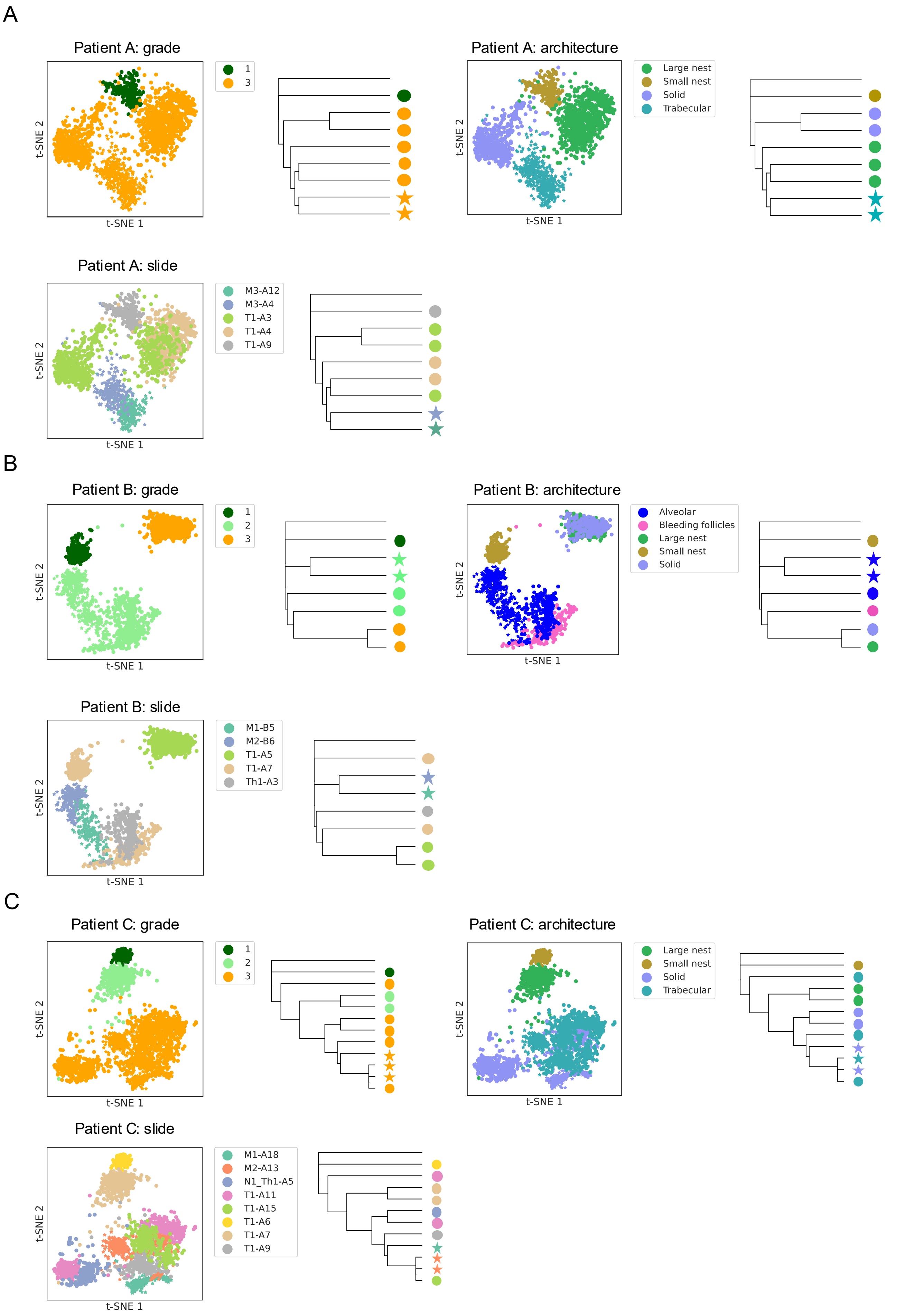

Fig. S11. A-C) Phylogenetic trees as in Fig. 4 in phylogram form with maintained edge lengths.

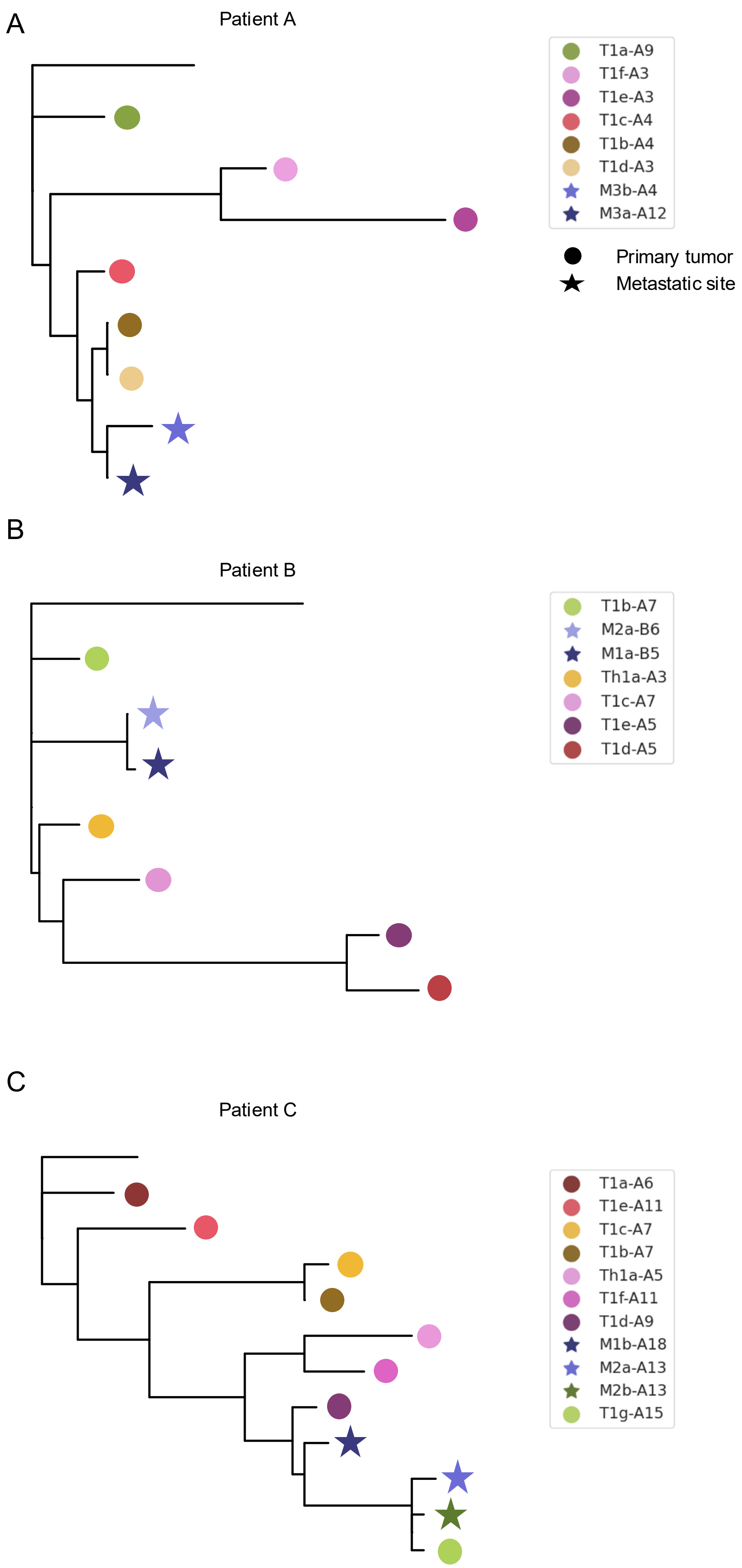

Fig. S12. A-C) Separation measure between all samples within patients A-C (1st column), with phylogenetic trees with highlighted clades containing samples that have low separation in morphology (lower half of the separation range, up to 3 samples; 2nd column).

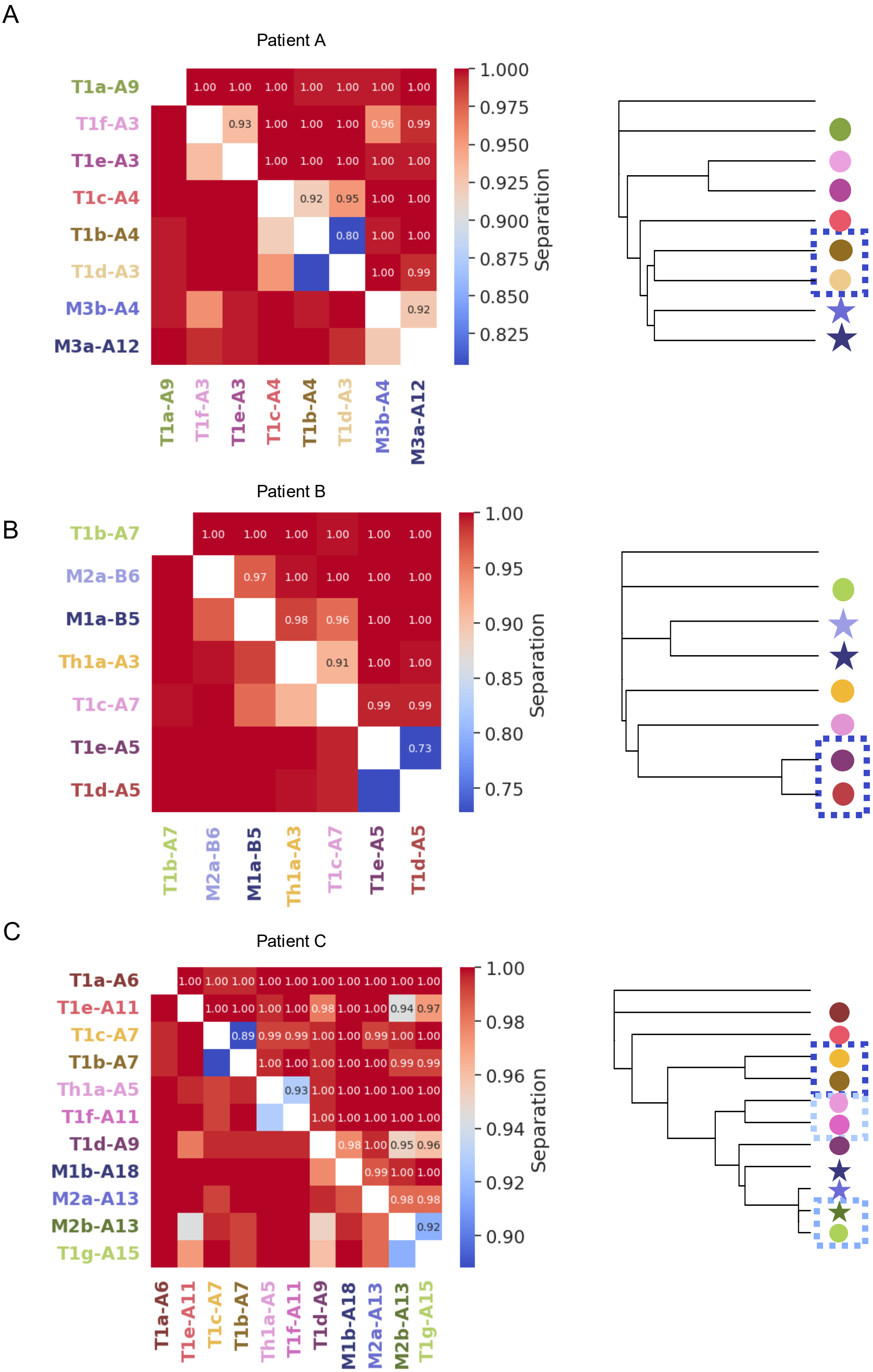

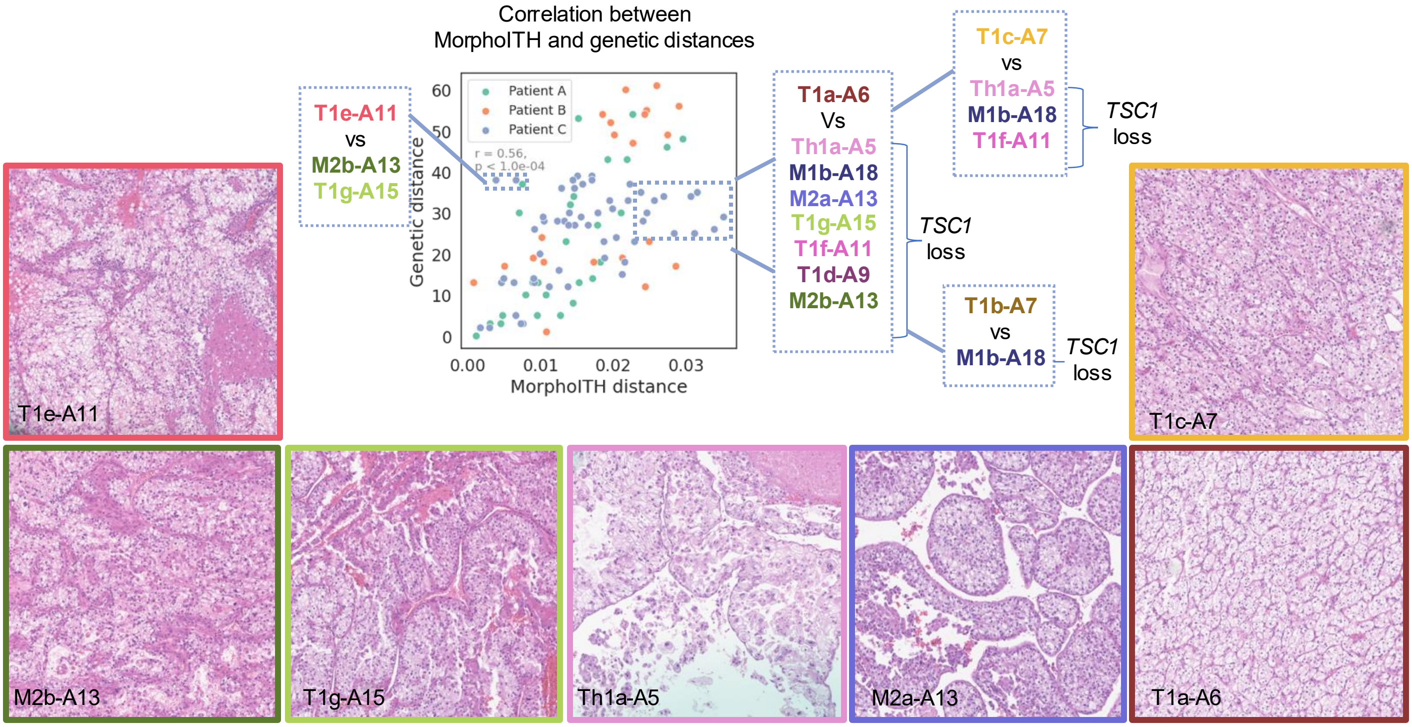

Fig. S13. Examples of outliers from the plot show correlation between MorphoITH (x-axis) and genetic (y-axis) distances from Fig. 4D. On the left, we show examples of sample pairs that are considered similar morphologically and less so genetically (T1e-A11 vs [M2b-A13, T1g-A15]). On the right, we show examples of sample pairs that are considered different morphologically but more closely related genetically (T1a-A6 vs [M2a-A13, Th1a-A5, T1g-A15, T1d-A9, M2b-A13, M1b-A18, T1f-A11], T1c-A7 vs [Th1a-A5, M1b-A18, T1f-A11], T1b-A7 vs M1b-A8).

Fig. S14. Per-patient correlation between MorphoITH and genetic distances on: A) original patches, or patches with applied normalization scheme as following: B) Macenko, C) Vahadane. Each plot includes Mantel's correlation coefficient with p-value.

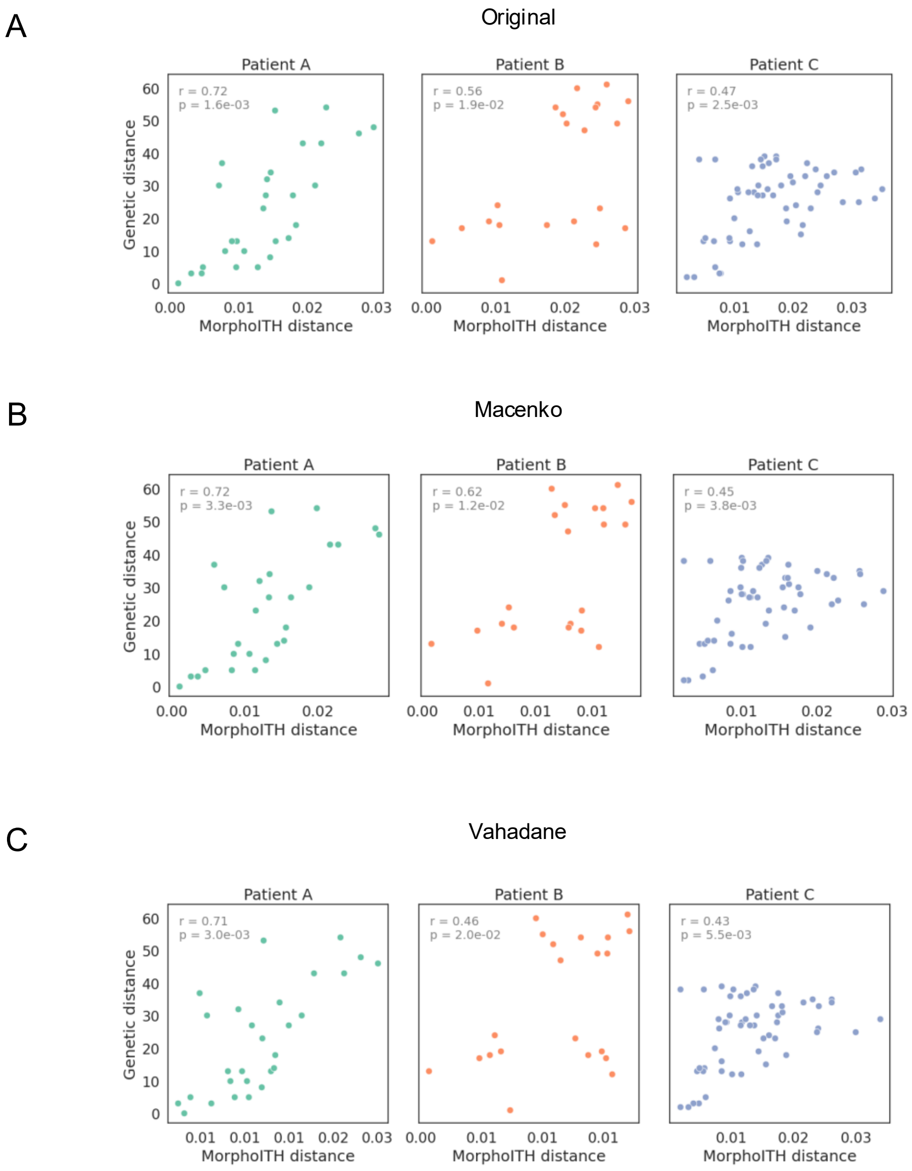

Fig. S15. Alternate Encoder for MorphoITH. A) Comparison of different models on the pretext retrieval task, in which we calculate the fraction of patches (y-axis) for which the N-th nearest neighbors (x-axis) were from the same TMA core (on held out portion of TMA Training). "ViTb / COMBINED" is the initial encoder of MorphoITH framework (trained on approximately 2/3 of the TMA Training dataset). We note that CONCH is designed for bigger patch size than the rest of the models. B) Scatterplot comparing MorphoITH-derived versus UNI-derived morphological distances for identical pairs of patches. Each point represents a randomly selected pair of patches from the WSI-3 cohort; its x-coordinate is the cosine distance between the pair using MorphoITH encoder features, and its y-coordinate is the cosine distance using UNI (v1) encoder features. Correlation is Pearson's r. C) Scatterplots comparing separability framework outputs from the MorphoITH encoder (x-axis) versus the UNI encoder (y-axis) across various biological comparisons from the WSI-3 cohort (derived from tasks analogous to those shown in Fig. 3B). Each point represents a specific biological comparison (e.g., a WT vs. Loss region pair for a given gene on a slide). Left, middle, and right panels show results for the Ground Truth, Contiguity Baseline, and Negative Control separability scores, respectively. Pearson correlation coefficients (r) are displayed on each plot. The dotted red line indicates y=x. Note: While UNI often yields systematically higher absolute separability scores (points generally above the y=x line), the strong correlations for both actual biological separability and the control baselines demonstrate that the final baseline-normalized classification of separability (full, partial, or none) remains highly consistent between encoders.

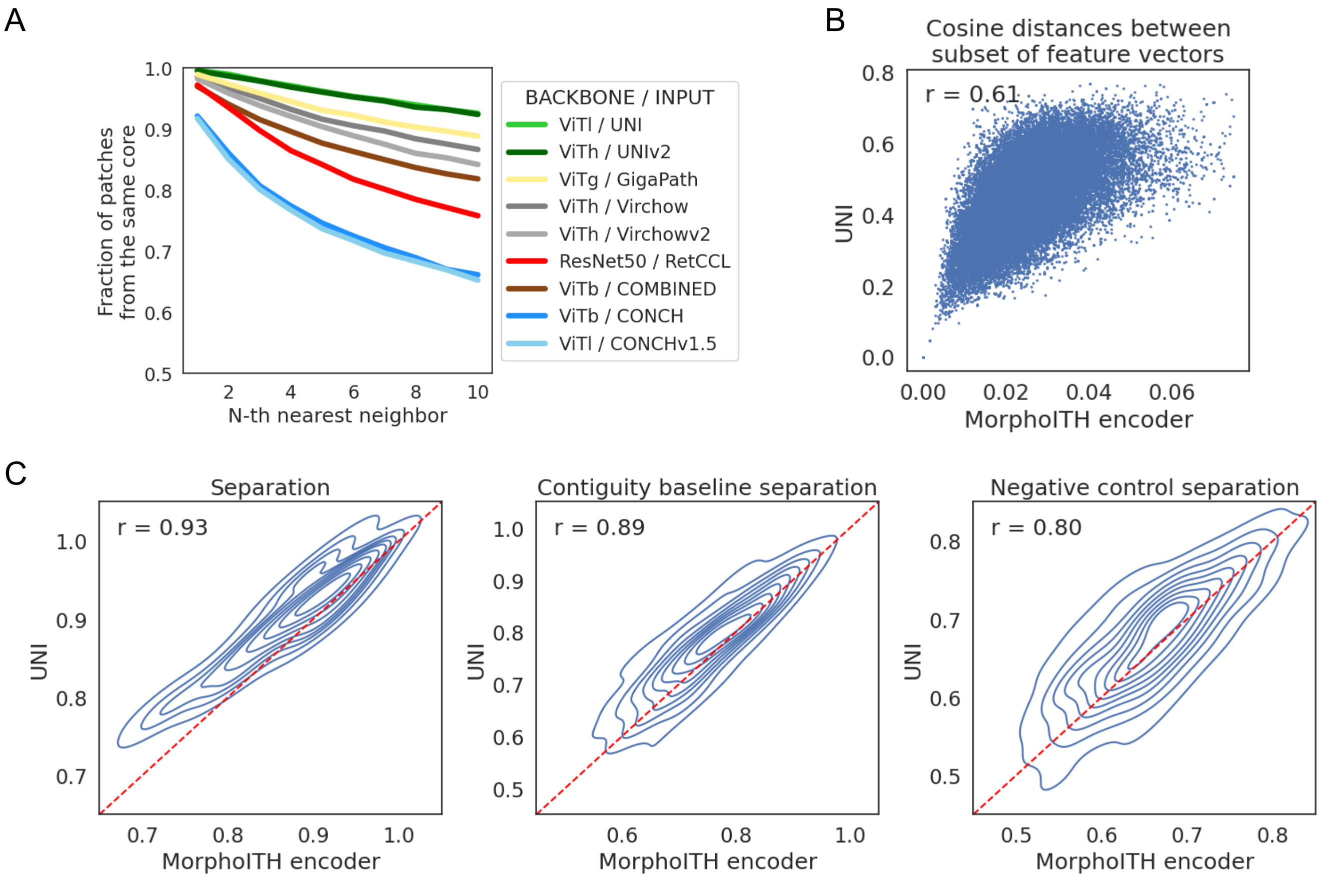

Fig. S16. Comparison between results of separation analyses as done using MorphoITH encoder (left) and UNI (v1; right). Separation of: A) Tumor vs non-tumor, B) architecture classes, C) nuclear grade (low vs high) separation, D) gene wild type and loss regions, E) architectures, F) nuclear grades (1-4).

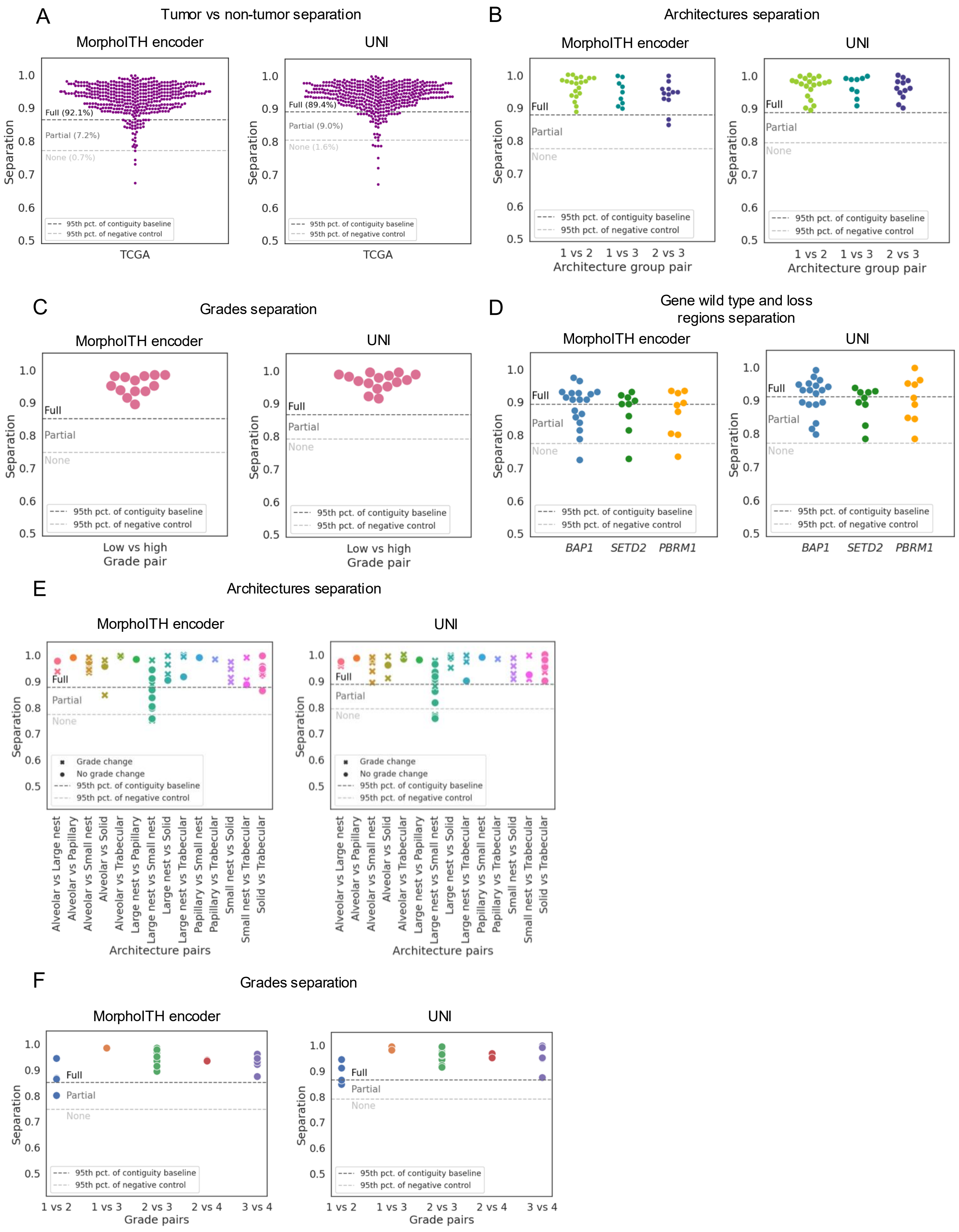

Fig. S17. A) Comparison between the Kaplan-Meier survival analysis in TCGA KIRC stratified by median heterogeneity score using MorphoITH encoder (left) and UNI (v1; right). B) Comparison between the global correlation between genetic distance (y-axis) and morphological distance (x-axis) for all patients using MorphoITH encoder (left) and UNI (right). Reported are the Mantel's correlation coefficients with their p-values.

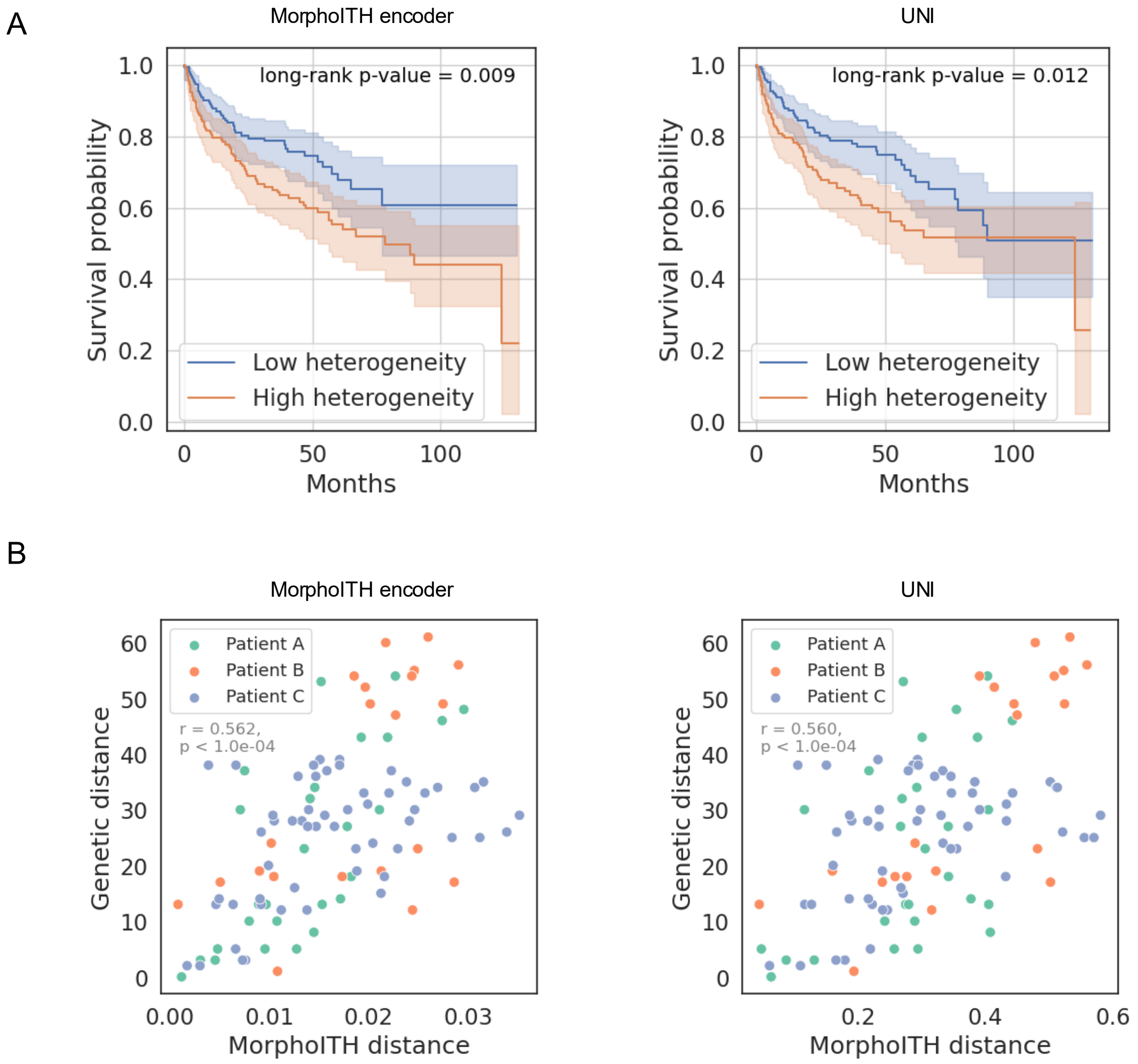

Fig. S18. A) Morphology guided sequencing (Fig. 3E) recreated with UNI (v1) as an encoder. B) For *BAP1* focal cases (1st column), we show focal ground truth (2nd column, loss region annotated in black), and morphological clusters (N=2) as derived from MorphoITH encoder (3rd column) and UNI (4th column).

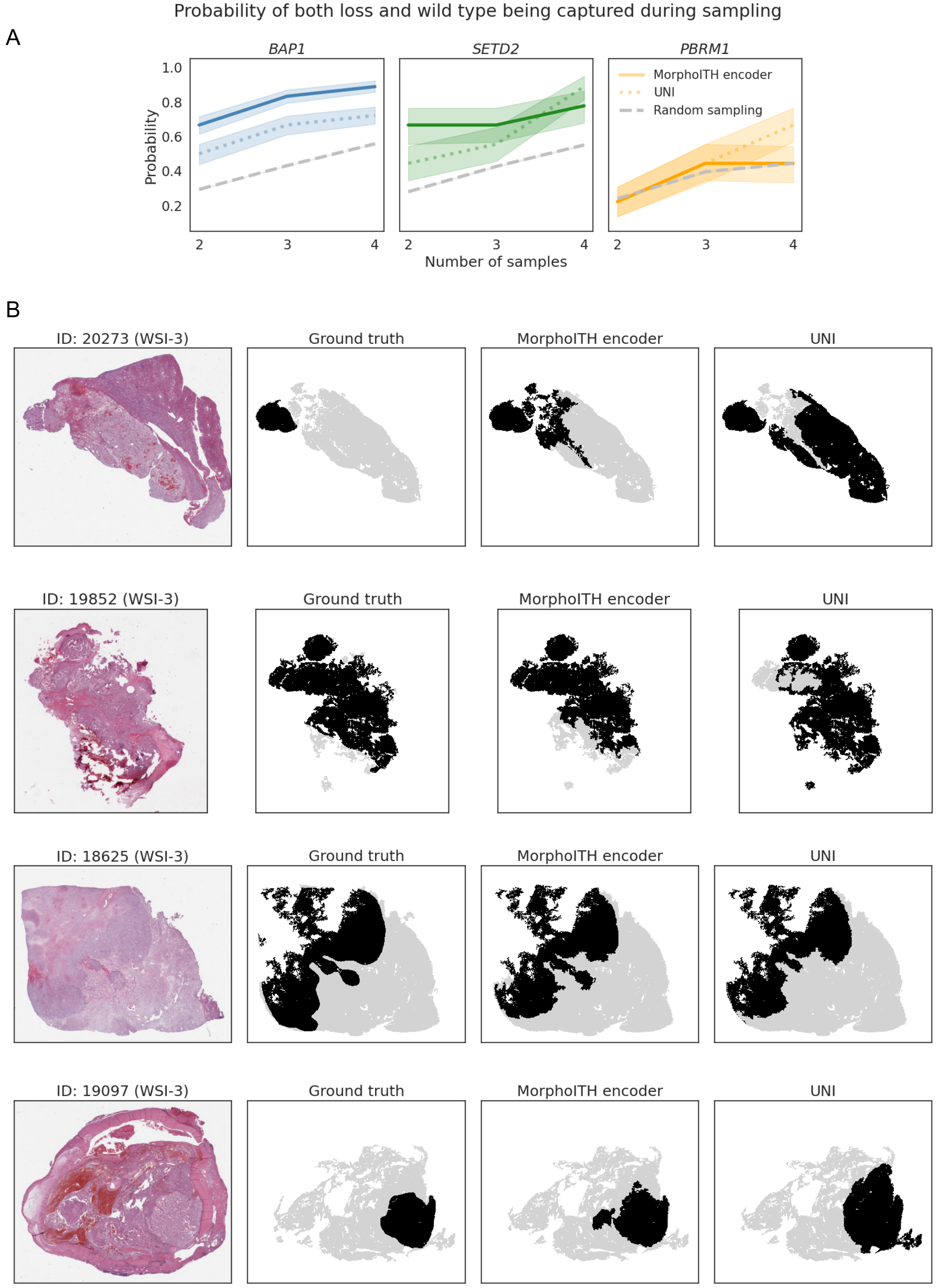

Supplement: Supplementary file 1 — Additional file 1. [file 13073_2025_1504_MOESM1_ESM.pdf]
